# Listeners' and Performers' Shared Understanding of Jazz Improvisations

**DOI:** 10.3389/fpsyg.2016.01629

**Published:** 2016-11-02

**Authors:** Michael F. Schober, Neta Spiro

**Affiliations:** ^1^Department of Psychology, New School for Social Research, The New SchoolNew York, NY, USA; ^2^Research, Nordoff Robbins Music TherapyLondon, UK; ^3^Faculty of Music, Centre for Music and Science, University of CambridgeCambridge, UK

**Keywords:** audience, improvisation, shared understanding, performance, listener, jazz, music cognition, interpretation

## Abstract

This study explores the extent to which a large set of musically experienced listeners share understanding with a performing saxophone-piano duo, and with each other, of what happened in three improvisations on a jazz standard. In an online survey, 239 participants listened to audio recordings of three improvisations and rated their agreement with 24 specific statements that the performers and a jazz-expert commenting listener had made about them. Listeners endorsed statements that the performers had agreed upon significantly more than they endorsed statements that the performers had disagreed upon, even though the statements gave no indication of performers' levels of agreement. The findings show some support for a *more-experienced-listeners-understand-more-like-performers* hypothesis: Listeners with more jazz experience and with experience playing the performers' instruments endorsed the performers' statements more than did listeners with less jazz experience and experience on different instruments. The findings also strongly support a *listeners-as-outsiders* hypothesis: Listeners' ratings of the 24 statements were far more likely to cluster with the commenting listener's ratings than with either performer's. But the pattern was not universal; particular listeners even with similar musical backgrounds could interpret the same improvisations radically differently. The evidence demonstrates that it is possible for performers' interpretations to be shared with very few listeners, and that listeners' interpretations about what happened in a musical performance can be far more different from performers' interpretations than performers or other listeners might assume.

## Introduction

When we attend a live musical performance or listen to a recording of one, to what extent do we understand what is happening musically—overall and moment-by-moment—in the same way as the performers do? And to what extent do we understand what is happening musically in the same way as our fellow listeners do?

Many performers probably hope that their listeners will pick up on the moments they see as having particular effects: being tender or shocking or climactic or virtuosic or a recapitulation. Many performers may also expect that their listeners—at least those in the know—will be able to recognize their actions: when they are particularly in sync, or when one of the performers takes the lead, initiates a new idea, or gives a cue to end. Listeners—perhaps especially those with playing experience (Pitts, 2013) or genre-specific listening experience—may similarly expect that they share substantial understanding with performers about musical structure or affect, or what they observe about the interaction among the musicians. As Radbourne et al. (2013a, p. xiii) put it, “the audience and performer crave a connectedness so that creativity is shared.” Audience members (whether they are physically copresent or listening alone) can also value “collective engagement” or “connection”: the feeling that they are experiencing the same emotions and having the same thoughts as their fellow audience members (Radbourne et al., 2013b; Zadeh, in press).

But to what extent do listener interpretations actually overlap with those of the performers and other listeners? Much less is known—scientifically or by performing arts organizations hoping to understand and build their audiences—about listeners' experience (what they think, feel and do during and after a performance) than about the demographic characteristics of audiences going to concerts or buying recordings (Radbourne et al., 2013a; see also chapters in Burland and Pitts, 2014). Investigations of differences in actor-observer experience and cognition (Jones and Nisbett, 1972; Malle et al., 2007, among many others) and important studies of musical communication (Williamon and Davidson, 2002; Hargreaves et al., 2005; Davidson, 2012; Loehr et al., 2013; Cross, 2014; Keller, 2014; Bishop and Goebl, 2015, among many others) have not directly focused on how performing musicians' ongoing mental life while performing connects and doesn't connect with listeners' ongoing mental life as they experience the performance. We see these questions as addressing broader and fundamental unknowns: when and with whom do our thoughts and/or feelings overlap, and when and with whom do they not—whether or not we think they do?

Our aim in this study is to look directly at the overlap in interpretations between listeners and performers in a new way: by examining the extent to which listeners to recorded live improvisations on a jazz standard (in this case by performers playing together for the first time) have the same interpretations of moment-by-moment and general characterizations of those improvisations that the performers themselves have. Our starting point is performers' own characterizations, as elicited in intensive yet quite open-ended one-on-one interviews, in the spirit of Schütz's (1951) arguments about the importance of performer experience in understanding collaborative music-making. The material thus ranges across topics the performers thought worth mentioning, rather than focusing on specific predetermined topics (e.g., music-analytic characteristics, performers' intentions, emotional responses, or details of their own or other's playing) or prioritizing particular kinds of understanding (perceptions, thoughts, feelings, judgments, characterizations, interpretations, etc.).

With this starting point, we ask whether listeners endorse characterizations that the performers agree about, and disagree with characterizations the performers disagree about; whether they agree with the performers' judgments any more than they agree with a commenting listener's judgments; and whether some listeners—those with greater musical or genre expertise—are more likely to agree with the performers than other listeners do. Our focus is on listeners' understanding during solo listening to an audiorecording of a live performance, rather than how listeners experience a live (or audio- or videorecorded) performance in which they are physically copresent with and can be affected by the reactions of other listeners as an audience in a shared space (e.g., Pennebaker, 1980; Mann et al., 2013; Koehler and Broughton, 2016; Zadeh, in press). Because listeners are not copresent with the performers nor do they see video of the performers, additional factors that can affect audience experience, like eye contact between performers and audience members (e.g., Antonietti et al., 2009) or visual characteristics of the performers (Davidson, 2001, 2012; Thompson et al., 2005; Mitchell and MacDonald, 2012; Morrison et al., 2014), do not come into play.

### Competing views

Common-sense intuitions about the nature of performance and audience-performer interaction offer up a number of competing possible answers to our research questions. On one extreme, as we see it, is *radical idiosyncrasy*: experiencing music is so idiosyncratic and personal, with no single “correct” hearing, that no two listeners will ever have identical experiences and interpretations. If so, the likelihood that any single listener's understanding overlaps with a performer's, or with any other listener's, should be low. This view is consistent with the fact that different music critics can have opposite reactions to the very same live performance or recording, and concert audiences and record buyers can range enormously in what they think of the same performances and recordings—and which concerts they choose to attend and which records they buy. It is also consistent with Kopiez's (2006, p. 215) observation that one view of musical meaning is that listeners always listen to music with “their own ears.”

A less extreme position is *minimal overlap*: listeners may share understanding with the performers or each other about the music's rhythmic structure or basic musical features, but further interpretations and evaluations could be entirely idiosyncratic. This view is implicit in claims that music has no meaning, or that any meaning that might be communicated is in terms of musical structure or basic but non-specific emotion or general affect (see Kopiez, 2006, for discussion). It is also consistent with the argument that musical shared experience simply involves entraining to the same stimulus, that music can have “floating intentionality” that is context-bound rather than exclusively communicating stable precise meaning (Cross, 1999, 2014), and with the view that music is not for communication of specific meanings or emotions but for synchronization of brains (Bharucha et al., 2011).

Yet another set of views—*specific-content* views—see musical performance as involving specific actions and musical products that listeners may “get”—or not. Musical interaction can involve give-and-take between performers (e.g., King, 2006; Keller, 2014) that audience members may pick up on, and the music produced has the potential to allow interpretation of *particular* affect, expression, and musical structure (e.g., Hargreaves et al., 2005). Audience members who are paying attention and have similar enough background knowledge to understand the music will pick up on which moments are (and perhaps that the performers also intend to be) tender or shocking or climactic or virtuosic or a recapitulation. Audience members in the know will also recognize performers' actions: when they are out of sync, or when one of the performers takes the lead, or initiates a new idea, or gives a cue to end. They can also share background cultural knowledge of the broader context that informs musical meaning (Clarke, 2005). This set of views is consistent with the fact that critics and audiences can have substantial consensus about the nature and quality of particular performances or performers, even if some listeners disagree.

Under any views that assume that *something* is communicated or interpretable, it makes sense to hypothesize that listener experience will be critical: Listeners with experience listening to classical Indian ragas or jazz standards or hip-hop should attend to the music, hear distinctions and understand musical gestures more as the performers do than listeners with no experience in the genre (see Zadeh, in press, for ethnographic evidence on how more expert listeners to Indian ragas publicly react differently than less expert listeners). It also makes sense to hypothesize that *degrees* of experience should predict the degree of overlap (a *more-experienced-listeners-understand-more-like-performers* hypothesis). Listeners who have themselves performed within a genre may well overlap with performers' understanding more than non-performers—they may have what Pitts (2013) called a “heightened experience of listening.” Perhaps, even, listeners who play the same instrument as a performer (independent of genre) might overlap more in understanding with *that* performer, compared with any co-performers who play different instruments (Pitts, 2013). This would be predicted if experienced performers understand music they are listening to by mentally simulating what performers do (Keller et al., 2007).

If one considers musical interaction as a form of joint action more generally, a quite different *listeners-as-outsiders* view is also plausible. Theorists of social behavior have argued that outsiders to an interaction (nonparticipants, observers, bystanders, eavesdroppers) can experience and understand what is happening differently than participants (interactants, speakers and addressees) (e.g., Goffman, 1976; McGregor, 1986; Clark, 1996). Empirical evidence supports this distinction in at least one non-musical domain, spoken dialogue: overhearers of live and audiorecorded dialogues about unfamiliar figures tend to understand participants' references (e.g., “the figure skater,” “the rice bag,” “the complacent one”) less well than participants do. This happens even when participants are strangers talking about figures that they have never discussed, and so they are unlikely to share expertise that would exclude overhearers (Schober and Clark, 1989).

Of course, music performance is different from conversation: it doesn't usually have referential content or the kinds of transactional goals that form the basis of many conversations (Cross, 2014), performers can produce sound at the same time as each other for extended periods (overlap in conversation, though common, tends to be brief, e.g., Schegloff et al., 1974; Stivers et al., 2009), and performers often design their music-making to be heard by non-participants (while many conversations are intended to be private). In live performance settings, audience members can also produce sounds that become part of the sonic landscape of the performance (Schober, 2014). Nonetheless, in improvisations on a jazz standard of the sort we are considering, the role and nature of the contributions by performers and listeners are substantially different in ways akin to the ways that interlocutors' and outsiders' roles and contributions differ in conversation, and so perhaps similar distinctions in perception and comprehension occur.

Collaborating musicians can certainly have the strong feeling of privileged intersubjective understanding: that what they shared in performing was unique to them and could not have been experienced by an outsider (see Sawyer's, 2008 descriptions of the phenomenon of “group flow”). This tacit belief was expressed by one player in a duo (Schober and Spiro, 2014) when he argued that a characterization of one of his improvisations that he disagreed with must have been made by an outsider rather than his co-performer, though that wasn't the case. But musicians can have competing views on how unique performers' perspectives are, relative to outsiders; as pianist Franklin Gordon (Sawyer, 2008, pp. 55–56) put it, “…at some point when the band is playing and everyone gets locked in together, it's special for the musicians *and for the aware conscientious listener* [emphasis added]. These are the magical moments, the best moments in jazz.” This statement assumes that attentive listeners with relevant expertise can join in performers' assessments and experience.

### Research approach and questions

The current study approaches these questions about audience perceptions by starting from an observation: when jazz improvisers independently characterize a joint performance of theirs in words, they may choose to talk about different moments or aspects of the performance, and they may have different judgments about even the same moments (Schober and Spiro, 2014; Wilson and MacDonald, 2016). When faced with their performing partners' characterizations, even though they can agree with much of what their performing partner says, they can also disagree in important ways about what happened—for example, having different understandings of which player was responsible for a gesture at a particular moment, of music-analytic characteristics of the improvisation, and of how much they enjoyed the performance. They can also endorse a commenting listener's characterizations of the performance more than they endorse their partner's (Schober and Spiro, 2014).

We start with the characterizations of three improvisations on a jazz standard from Schober and Spiro (2014) individually generated by the players in a saxophone-piano duo and a commenting listener (himself an experienced jazz saxophonist), for which we have performers' ratings of endorsement and for which we know when their ratings agreed with each other's. In order to elicit responses to (at least one version of) what the performers themselves thought about the improvisation, we start with these statements rather than evaluative jury ratings (e.g., Thompson and Williamon, 2003; Wesolowski, 2016), judgments of performer appropriateness (e.g., Platz and Kopiez, 2013), listeners' agreement with professional music critics' assessments, listeners' own descriptions of their reactions to music (e.g., Richardson, 1996; Heimlich et al., 2015, among many others), or listeners' ratings of their arousal or affective responses (e.g., Lundqvist et al., 2008; Eerola and Vuoskoski, 2013; Olsen et al., 2014, 2015; Olsen and Dean, 2016; and chapters in Juslin and Sloboda, 2010). Unlike the statements in standardized jury evaluations, a number of the statements used here focus on specific moment-by-moment characterizations of particular performances that were given in reference only to these performances.

Using these statements, we ask the following research questions about how listeners endorse the characterizations:

**Research Question 1:** Will listeners endorse statements that both performers endorsed more than statements the performers disagreed about (one endorsed and the other did not)?
**Research Question 2:** Will listeners with more genre expertise endorse performers' statements more than listeners without genre expertise?
**Research Question 3:** Will listeners who play the same instruments as the performers (saxophone and piano) endorse performers' statements more than listeners who do not play those instruments?

We also ask the following research questions about how listeners' patterns of judgment—as measured by their ratings across multiple statements—align with the performers' and commenting listeners' patterns of judgment:

**Research Question 4:** Will listeners agree with performers' judgments any more than they agree with a commenting listener's judgments?
**Research Question 5:** Will listeners with more genre expertise agree with performers' judgments more than listeners without genre expertise?
**Research Question 6:** Will listeners with expertise on a particular instrument agree more with judgments by a performer who plays that same instrument?

The pattern of results we observe will provide evidence that is consistent with, or rules out, competing views and hypotheses about listeners' shared understanding with performers and with each other, from *radical idiosyncrasy* to *minimal overlap* to the *more-experienced-listeners-understand-more-like-performer*s and *listeners-as-outsiders* hypotheses. We explore these views in two ways, looking both at levels of endorsement of statements and agreement across listeners (patterns of ratings across multiple statements). For endorsement, if all listeners (or all listeners with a certain kind of expertise) were to endorse or reject a set of characterizations, we can take that as evidence of consensus among the listeners. A split among the listeners would suggest a lack of shared understanding. For agreement, if any two listeners were to have the same ratings (in the direction of endorsing, rejecting, being neutral, or not understanding) on every single statement in the study, we would take this as evidence of substantial shared understanding, though of course the statements included in the study are not exhaustive of everything a listener might understand about a performance. If two listeners differ in their judgment on every single statement in the study, we would take this as evidence of substantial disagreement.

## Methods and materials

This study was carried out following ethical review by the Institutional Review Board at The New School (Approval #97-2013) and endorsement of that review by the Research Ethics Committee at Nordoff Robbins Music Therapy. All participants provided informed consent in accordance with the principles in the Belmont Report and the New School Human Research Protections Program.

### Materials

The audio files that formed the basis of listeners' judgments were the three recorded improvisations by a sax player and pianist on “It Could Happen to You” used in Schober and Spiro (2014). (Audio files are available under Supplementary Material). All data collection in the current study occurred before the publication of the Schober and Spiro (2014) paper, so there was no chance that listeners could have been affected by any interpretations in that article nor that they could have been aware of the sources of the characterizations they were rating.

### Questionnaire

A questionnaire consisting of three main sections was developed. Two sections consisted of statements that listeners were to rate on a 5-point scale with labels “strongly disagree,” “disagree,” “neither agree nor disagree,” “agree,” and “strongly agree,” with the additional option of “don't understand” for each statement. A third section asked detailed questions about listeners' own musical experience.

The statements to be rated in the first section were 24 anonymized statements that had been made by the performers and a commenting listener in independent post-improvisation think-aloud interviews, chosen from the 151 unique statements characterizing the three improvisations in Schober and Spiro (2014). In those interviews, the performers and commenting listener had been prompted with questions about both performers' intentions, what worked and didn't work in the performances, and their general and moment-by-moment characterizations of what had happened, first from memory and then prompted by interviewee-controlled listening (potentially multiple times) to the recordings.

The 24 statements were selected on the basis of three major criteria. First, the set of statements was to include an equal number of statements (12) that both performers had agreed about (either both rated 4 or 5 on the 5-point scale from “Strongly Disagree” to “Strongly Agree,” or both rated 1 or 2) and statements that they had disagreed about (one agreeing and the other disagreeing, that is, ratings that crossed the “neutral” divide). This was so that we could see whether listeners in the current study would be more likely to endorse statements that the performers had originally both endorsed (Research Question 1). Second, the set of statements was to include an equal number of statements (8) that had originally been made by the sax player, the pianist, and the commenting listener, to test whether listeners might “side” with statements made by one party more than others. Third, there were to be roughly equal numbers of statements about each of the three improvisations, so that listeners would consider several specific characterizations of each of the three (quite different) improvisations.

Within these constraints, statements were randomly selected from the pool of available statements with two additional constraints: removing highly technical statements that would likely exclude listeners without significant music theory training (e.g., “At about 0:17 the piano changes the quality to Phrygian, signaling a more functional dominant”), and disqualifying any statements that could only be understood in reference to a previous statement, or including the relevant prior statements if they also satisfied the other criteria (items 8–10 about the “turnaround” in Table 1, which lists the items in the order in which listeners encountered them). Because the final set of statements included a few technical terms that could potentially be unfamiliar to listeners with no formal musical training (e.g., “chorus,” “substitutions,” “turnaround,” “vamp”) brief definitions were selected for each of these terms so that they could be included in the questionnaire (all terms with asterisks in Table 1), and one additional item that provided context for the second item and that defined “chorus” was inserted so that listeners without formal training would be able to rate item 2 as well as subsequent items using the word “chorus.”

**Table 1:** **The performance-specific statements about which listeners rated their agreement, in the order of presentation**.

**ID**	**Statement**	**From recording**	**Originator**	**Performer agreement**
1	The overall performance was standard or “vanilla.”	1	Sax	Agreed
2 (context—not for analysis)	Starting at about 1:22, the sax takes two choruses^*^. ^*^chorus: a part of a song that recurs at intervals, usually following each verse; refrain. (from Dictionary.com)	1	Commenting listener	n/a
2	During these two choruses starting at about 1:22 the sax hears and uses the pianist's substitutions^*^. ^*^Chord substitution is the technique of using a chord in the place of another, often related, chord in a chord progression. Jazz musicians often substitute chords in the original progression to create variety and add interest to a piece. (from Wikipedia)	1	Commenting listener	Disagreed
3	When the pianist played in the same range^*^ as the sax at about 1:37, the pianist was stepping on the sax's toes. ^*^The same general span of notes (same general pitch height)	1	Pianist	Disagreed
4	When the pianist played a solo line over the sax from 1:53 to 1:59, the pianist was stepping on the sax's toes.	1	Pianist	Disagreed
5	At about 4:39 end of piano solo, the pianist played the same chord that the sax played at the end of the sax solo.	1	Pianist	Agreed
6	The pianist gave a cue to end at about 6:00 by using the pedal.	1	Pianist	Disagreed
7	At about 6:10, the sax plays a classic wrap-up cliché.	1	Sax	Agreed
8	At 1:57 to 2:03 the sax plays a turnaround^*^ at the end of the melody to get back to the top of the sax solo. ^*^In jazz, a turnaround is a passage at the end of a section which leads to the next section. This next section is most often the repetition of the previous section or the entire piece or song (from Wikipedia)	2	Commenting listener	Disagreed
9	At 1:57 to 2:03 the piano does not pick up the turnaround.	2	Commenting listener	Agreed
10	At 1:57 to 2:03 because the piano does not pick up the turnaround, things are a bit discombobulated between the two players.	2	Commenting listener	Disagreed
11	At about 2:05 the players find the top^*^ together and are OK again. ^*^The beginning point of each chorus, the first beat of the first measure (definition by Darius Brotman)	2	Commenting listener	Disagreed
12	From about 2:40, the sax signaled the end of one chorus and the beginning of the next.	2	Pianist	Agreed
13	At about 2.50 there was nice and memorable interplay.	2	Sax	Agreed
14	The pianist continued the sax's phrasing at about 2:55.	2	Pianist	Agreed
15	In the last phrase the sax played, the sax was “fishing” to get out of the tune.	2	Sax	Disagreed
16	The sax played a cliché ending at about 6:29.	2	Pianist	Agreed
17	This version took the most harmonic liberties.	3	Sax	Disagreed
18	This version had the most motion.	3	Sax	Disagreed
19	The pianist set the tempo.	3	Commenting listener	Agreed
20	The pianist's opening was excellent.	3	Sax	Disagreed
21	At about 1:38 the sax begins the sax's second chorus and the piano begins accompanying it.	3	Commenting listener	Agreed
22	During this chorus, the sax also plays a somewhat fragmented improv, and with lines that pull away from the harmony.	3	Commenting listener	Agreed
23	At about 4:52 the sax intended to play another chorus.	3	Pianist	Disagreed
24	The vamp^*^ ending was fun. ^*^A simple section like a riff, designed to be repeated as often as necessary (definition by Darius Brotman)	3	Sax	Agreed

*All “agreed” statements in this final set were endorsed by both performers, and in no case did the originator disagree with their original statement*.

The second section included 42 statements to be rated (the same 14 for each improvisation) about the general quality and character of each improvisation, adapted from a jury evaluation system used at The New School for Jazz and Contemporary Music. It included 7 additional global statements about the performers that had been generated by the performers and commenting listener (e.g., “The sax's signals are very clear”; “The pianist is open to doing whatever happens in the moment”). This section was included for analyses that go beyond the scope of this paper and will be reported elsewhere.

A final set of questions asked about listeners' musical background and experience (in general and in jazz), using and adapting questions from the Gold-MSI (Müllensiefen et al., 2014); their experience filling out the questionnaire; and about their demographic characteristics.

See Table 1 in Supplemental Data for the set of all questions asked in the study.

### Online survey implementation

The questionnaire was implemented in the Qualtrics platform for presentation in a web browser (Qualtrics, 2014)^1^, allowing participants to answer on their own devices at a time and place convenient for them and to take breaks if needed. We both screened participants and collected the data through the Qualtrics questionnaire.

After receiving a link to the survey (see Recruitment), participants first encountered a few screening questions and a consent form. Once they started the survey, they were instructed to only begin once they were in a place in which they could listen privately with good audio. Once they agreed that they were ready to begin, participants were presented with the audio file of the first improvisation, along with instructions that they were to listen once through entirely (at least, and more often if they liked) before starting to answer the questions. Listeners could start and stop audio presentation as they preferred, and listen as often as they liked. The survey software was programmed so as to prevent participants from proceeding to the questions until as much time as the recording took to play once through had passed. Each screen of survey questions about a performance included the complete audio file so that listeners could relisten as often as they liked while answering those particular questions. On each page the audio file was set to start, if the listener clicked, at the moment in the performance that the first question on that page was about, but the full performance was available for listening as desired^2^.

**Figure 1:**
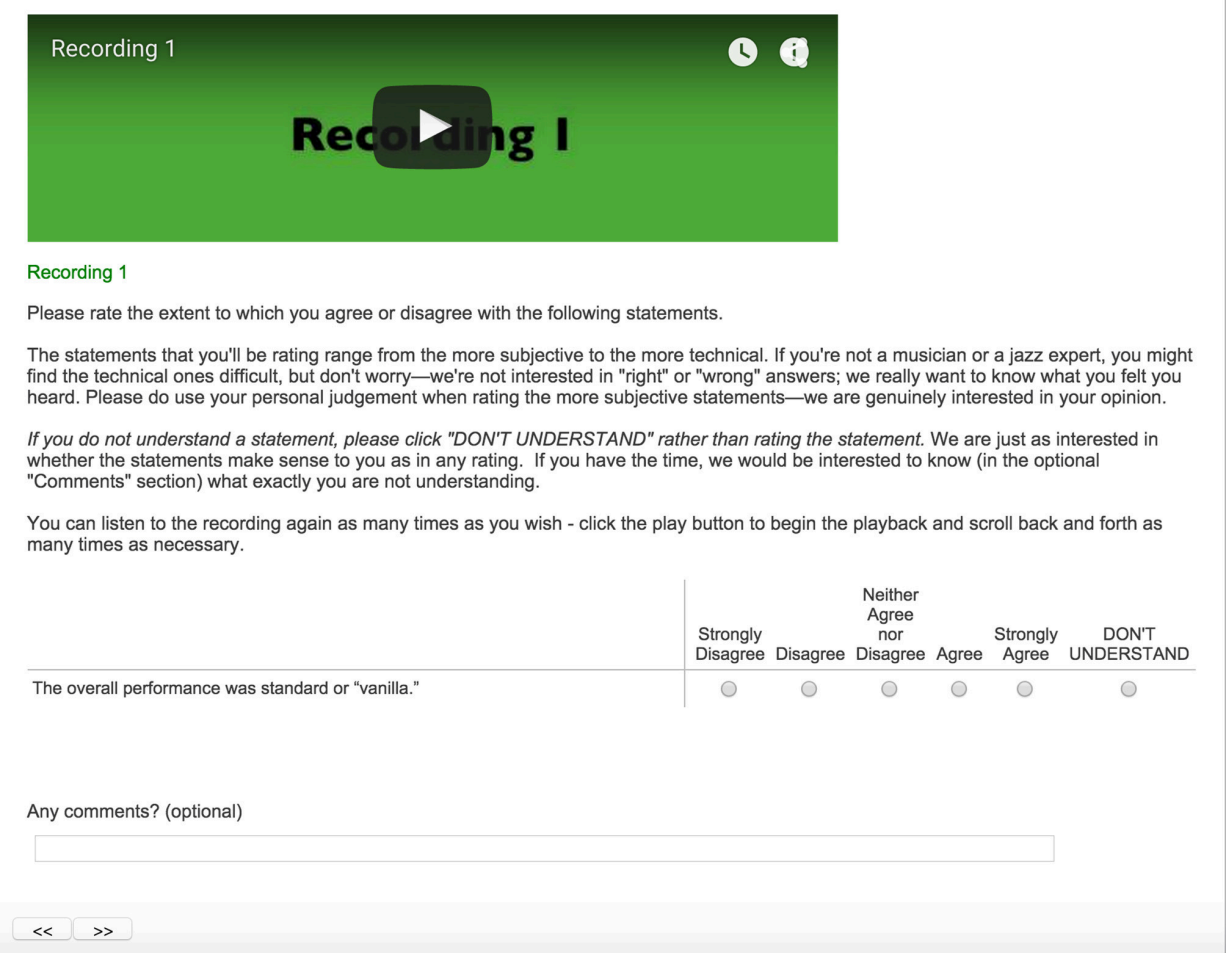
**Screen shot of the first statement to be rated, including additional instructions to listeners**.

**Figure 2:**
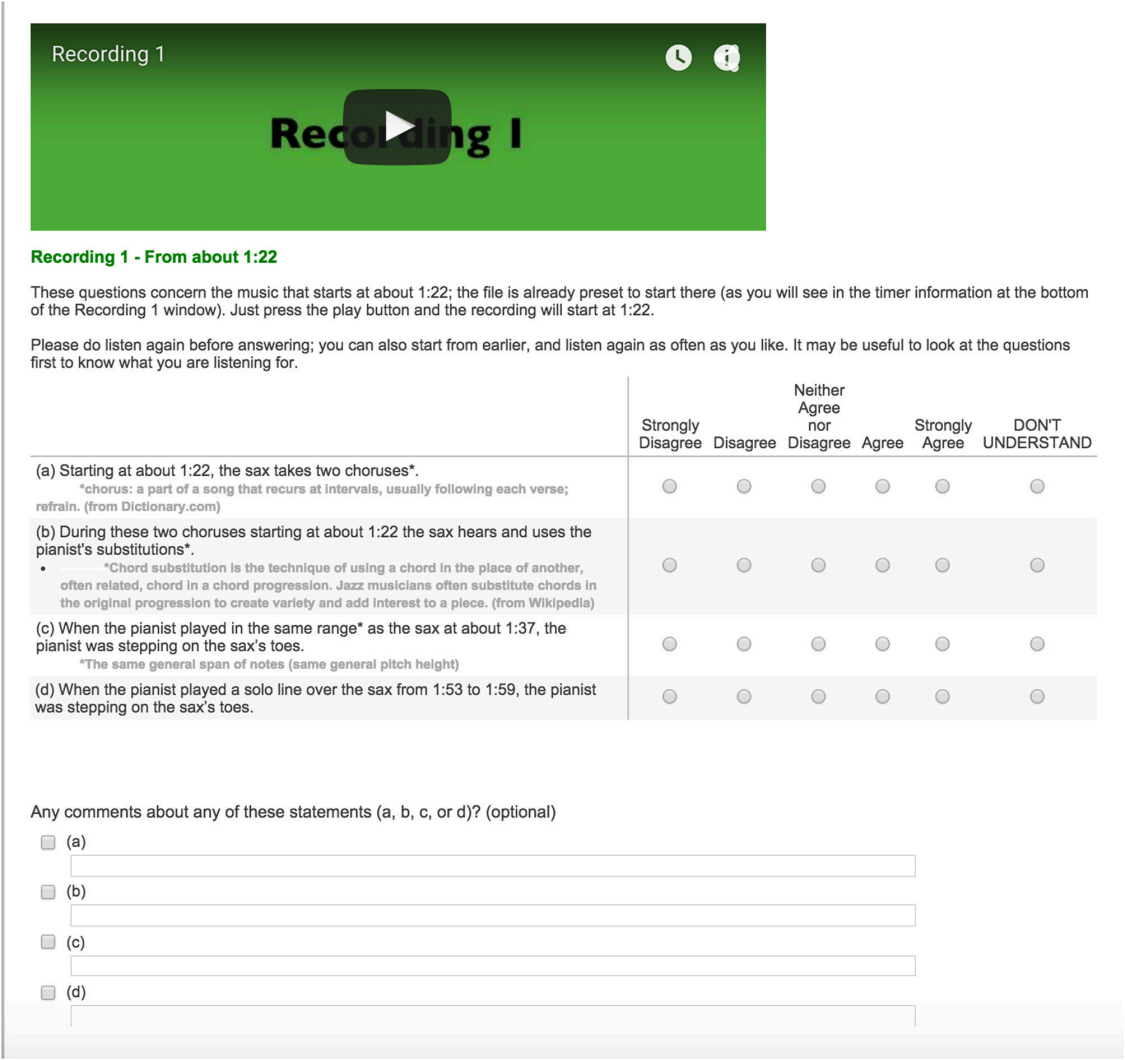
**Example screen shot with layout of embedded audio file, multiple statements to be rated, and the response options (“Strongly Disagree” to “Strongly Agree,” as well as “Don't Understand”) in the online survey**.

Figures 1, 2 show screenshots of the layout of the embedded audio files, statements to be rated, and the response options (“strongly disagree” to “strongly agree,” as well as “don't understand”). For each statement listeners could also write in optional comments. The same layout was applied for the second and third improvisations.

Based on informal usability testing, the task was expected to take about 1 h.

### Recruitment

Musicians (with jazz and non-jazz backgrounds) and non-musicians were recruited to participate in an intensive online study for a $20 Amazon gift card incentive, to be awarded on completion of the full study. The intention was to recruit as broad a spectrum of listeners—musicians and non-musicians, with jazz backgrounds and non-jazz backgrounds, living in a range of countries—as possible. Our recruitment procedure was inspired by Respondent Driven Sampling (RDS) methods, with direct email invitations sent to particular targeted “seed” participants from the authors' networks who belonged to the kinds of communities we were interested in as participants, although we did not do the more systematic analyses of participants' social networks that would be required for a full implementation of RDS (Heckathorn and Jeffri, 2003; Gile et al., 2015). Recruits were also invited to forward the email invitation to others they thought might be interested. In order to target the range of participants we were hoping for, the link in our email invitation sent recruits to a small set of initial screening questions in Qualtrics, asking whether they considered themselves to be musicians or not, and if so what genre they considered to be their primary genre and what instrument they played. A running tally of participants based on these screening categories (musician, non-musician, genre, instrument played) was intended to guarantee representation of a range of particular types of experience in our final sample, and to prevent overrepresentation of any one category.

Because of an unexpected post of the link to the study on the Reddit social media and discussion website several weeks after the survey was launched there was a sudden upsurge of interest, and so this screening procedure ended up not filtering out participants as we had intended; some potential participants ended up answering the screening questions differently several different times in order to gain access to the study. We filtered out and did not analyze the data from any participants who used this technique. For subsequent analyses, we relied on the much longer set of final questions about participants' musical background at the end of the questionnaire to categorize their experience, on the assumption that participants who had spent an hour on the study and provided careful and differentiated answers would answer the longer set of questions about their musical experience accurately.

### Data preparation

Our recruitment procedure and the available budget led to 285 completed cases, out of the 320 cases where participants answered the first substantive question in the survey (rating their agreement with the statement “The overall performance was standard or vanilla”). This is an effective completion rate of 89.1% among those participants who started the survey.

Because completing this task requires attentive listening and thought, as well a substantial time investment, we wanted to make sure to only analyze data from participants who had taken the task seriously. To assure this, we filtered out cases based on two additional criteria. (1) We filtered out all “speeders,” which we defined as anyone who finished the survey in under 30 min, as measured by Qualtrics log files. This made sense because simply listening to the three improvisations at all took 21:31 min. This removed 35 cases, which reduced our number of eligible cases to 250. (2) We filtered out any cases of “straightlining” (nondifferentiation) in the first section of the survey (the section that asked about specific moments in the music)—cases where a participant provided the same response option (from “Strongly Disagree” to “Strongly Agree,” or “Don't Understand”) for every question about a particular improvisation. This removed another 11 cases, which reduced our number of eligible cases to 239.

Interestingly, these removals do not change the overall pattern of results for participants' levels of endorsement at all, which is consistent with evidence from web surveys that removing the data from speeders doesn't necessarily change findings (Greszki et al., 2015). Nonetheless, this seemed an important precaution given that in web surveys people who speed are also more likely to straightline, suggesting that these behaviors come from a common tendency to “satisfice” (Zhang and Conrad, 2014).

### Participants

The 239 participants included in the final data set were almost all (87.4%, *n* = 209) self-reported musicians. More than half the participants (54.0%, *n* = 129) classified themselves as playing jazz (see Table 2); more than half (54.0%, *n* = 129) reported playing in more than one genre, and of the 129 jazz players only 27.1% (35) reported only playing jazz. 61.5% (147) reported listening to jazz regularly, with 53.1% (127) reporting listening to other genres in addition to jazz regularly. 65.7% (157) reported 5 or more years of daily playing, and 64.4% (154) reported at least some experience improvising. 68.2% (163) reported having ever played piano, 31.0% (74) reported having ever played saxophone, 20.5% (49) reported having played both piano and saxophone, and the rest had played other instruments. The majority (55.6%, *n* = 133) reported playing more than one instrument on a regular basis now, with 31.8% (76) reporting playing only one instrument on a regular basis. Almost all (93.3%, *n* = 223) reported “really loving” or “liking” music, with no participants reporting “really hating” or “disliking” music. 79.1% (189) reported “really loving” or “liking” jazz.

**Table 2:** **Musical experience of the 239 participants in the data set**.

	***n***	**%**
**Which genre(s) of music do you play? (select all that apply)**		
Rock/pop	104	43.5
Jazz	129	54.0
Classical	136	56.9
Folk	45	18.8
Other (write-ins included: blues, electronic, Korean pop, musical theater, RandB, reggae, electronic, metal, fusion, among others)	21	8.8
None	15	6.3
**Which genre(s) of music do you listen to regularly? (select all that apply)**		
Rock/pop	177	74.1
Jazz	147	61.5
Classical	146	61.1
Folk	75	31.4
Other (write-ins additionally included: Christian, experimental, hip hop, international, soul, Latin, among others)	29	12.1
None	3	1.3
**How much do you like music?**		
Neutral	14	5.9
Like	57	23.8
Really love	167	69.9
**How much do you like jazz?**		
Really hate	1	0.4
Dislike	7	2.9
Neutral	42	17.6
Like	112	46.9
Really love	77	32.2
**For how many years did you engage in regular, daily practice/playing of a musical instrument (including voice)?**		
0 years	5	2.1
Up to 2 years	24	10.0
2–5 years	53	22.2
5–10 years	78	32.6
10 or more years	79	33.1
**Which of these instruments do you now play on a regular basis? (select all that apply)**		
Saxophone	49	20.5
Other wind and brass	83	34.7
All wind and brass	132	55.2
Piano	130	54.4
Other keyboard	52	21.8
All keyboard	182	76.2
Percussion	43	18.0
Voice	84	35.1
Strings	142	59.4
Other (congas, cornet, djembe, melodica, recorder)	6	2.5
None	30	12.6
**Which of these instruments have you ever played on a regular basis? (select all that apply)**		
Saxophone	74	31.0
Other wind and brass	147	61.5
All wind and brass	221	92.5
Piano	163	68.2
Other keyboard	74	31.0
All keyboard	237	99.2
Percussion	67	28.0
Voice	93	38.9
Strings	186	77.8
Other (congas, cornet, djembe, melodica, recorder)	6	2.5
None	7	2.9
**How much experience do you have in improvising musically?**		
None	29	12.1
A little	56	23.4
Some	80	33.5
A lot	49	20.5
A great deal	25	10.5
**How long do you listen attentively to music per day?**		
I never listen	1	0.4
30 min or less	34	14.2
30–60 min	89	37.2
60–90 min	60	25.1
90 min or more	55	23.0
**How long do you listen attentively to jazz per day?**		
I never listen	36	15.0
30 min or less	119	49.8
30–60 min	47	19.7
60–90 min	26	10.9
90 min or more	11	4.6

As Table 3 details, the respondents were mostly from the US (75.7%), the UK (9.2%), and Canada (8.8%), with more men than women and a range of ages, educational attainment, and incomes. 60.7% of the participants reported being White, 6.3% Black, 19.2% Asian, and 7.5% Hispanic or Latino.

**Table 3:** **Demographic characteristics of the 239 participants in the data set**.

	***n***	**%**	**2008 NEA survey of US jazz concertgoers (%)**
**I identify myself as**			
Female	81	33.9	52.4
Male	157	65.7	47.6
Not reported	1	0.4	
**What is your current age?**			
18–24	46	19.2	11.9
25–34	61	25.5	17.4
35–44	71	29.7	17.1
45–54	41	17.2	24.4
55–64	15	6.3	18.4
65 or over	5	2.1	10.8
**I classify myself as (select all that apply): (different countries and groups use different labels, so please add your own if needed)**			
Arab	3	1.3	
Asian/Pacific Islander	46	19.2	
Black	15	6.3	12.5
Caucasian/White	145	60.7	77.5
Hispanic	17	7.1	6.8
Indigenous or Aboriginal	1	0.4	
Latino	1	0.4	
Multiracial	6	2.5	
Other (European, I don't, Iranian, mixed English/Chinese, Persian, Persian/Norwegian, South Asian, West Indian)	8	3.3	3.2
Would rather not say	5	2.1	
**The country I reside in is**			
United States	181	75.7	100
United Kingdom	22	9.2	
Canada	21	8.8	
Albania	1	0.4	
Australia	1	0.4	
Japan	1	0.4	
The Netherlands	1	0.4	
Not reported	11	4.6	
**The highest level of education that I have completed is**			
Less than High School	1	0.4	4.0
High School/GED	49	20.4	15.1
Undergraduate/Bachelors Degree (including performance)	98	41.0	60.6
Masters Degree (including performance)	65	27.2	20.4
Doctoral Degree (including performance)	16	6.7	
Other Professional Degree (e.g., JD, MD)	5	2.1	
Would rather not say	5	2.1	
**On a scale of 1 to 5, my comprehension of the English language is**			
1 Poor	4	1.7	
2	0	0	
3	1	0.4	
4	30	12.6	
5 Excellent	202	84.5	
Unreported	2	0.8	
**My annual household salary (including bonuses and commissions) in U.S. dollars is**			
$0 – $50,000	86	36.0	33.5
$50,001 – $100,000	67	28.0	33.6
$100,001 – $150,000	30	12.6	17.7
$150,001+	12	5.0	15.3
Would rather not say	44	18.4	

How similar is our sample of participants to audiences for jazz performances or recordings? Because so many of our listeners reported living in the US, the most relevant comparisons are to the US jazz listening public. According to the most recent NEA Survey of Participation in the Arts (National Endowment for the Arts, 2009), which found that 7.8% of the US population in 2008 attended at least one jazz event, the demographic characteristics of our participants are relatively similar to this nationally representative sample (see rightmost column in Table 3), although the proportion of males in our sample is higher, and our sample was a bit younger, less well off, and more likely to report not being White than jazz audiences in the US in 2008.

The fact that our listeners were so musically experienced may not be such an unusual phenomenon (Pitts, 2013, p. 88): in one chamber festival in the UK, 63% of 347 audience research participants reported having previously played or currently playing music, including the instruments at the performances they attended. Of course, our sample's including so many experienced musicians limits the generalizability of our findings to listeners with the characteristics of our sample; the extent to which our findings generalize to listeners with different characteristics or different motivation to participate in a demanding online survey is unknown.

## Results

### Endorsement of the performance-specific statements

As Tables 4, 5 show, none of the 24 performance-specific statements was universally endorsed, and different statements elicited substantially different levels of endorsement. For the statements the players had agreed about, levels of endorsement ranged from 44.6% to 86.4%, and for the statements the players had disagreed about, levels of endorsement ranged from 33.5% to 77.3%. The four most-endorsed statements (of the 24) were ones that the performers had agreed about, and the three least-endorsed statements were ones the performers had disagreed about. But listeners had low endorsement of some statements the performers had both endorsed, and high endorsement of some statements the performers had disagreed about.

**Table 4:** **Percentages of listeners endorsing each of the 12 performance-specific statements that the performers had agreed about, ranked from most to least endorsed**.

**Statement**	**About recording**	**Statement originator**	**Agree (%)**	**Neither agree nor disagree (%)**	**Disagree (%)**	**Don't understand (%)**
The pianist set the tempo	3	Commenting listener	86.4	7.0	6.2	0.4
At about 6:10, the sax plays a classic wrap-up cliché	1	Sax	81.4	8.3	8.7	1.7
The pianist continued the sax's phrasing at about 2:55	2	Pianist	80.6	10.7	7.0	1.7
At about 1:38 the sax begins the sax's second chorus and the piano begins accompanying it	3	Commenting listener	77.7	12.0	9.5	0.8
At about 2.50 there was nice and memorable interplay	2	Sax	73.6	11.6	14	0.8
During this chorus, the sax also plays a somewhat fragmented improv, and with lines that pull away from the harmony	3	Commenting listener	68.6	16.1	11.6	3.7
From about 2:40, the sax signaled the end of one chorus and the beginning of the next	2	Pianist	60.7	16.9	21.1	1.2
At about 4:39 end of piano solo, the pianist played the same chord that the sax played at the end of the sax solo	1	Pianist	59.9	14.5	22.7	2.9
The overall performance was standard or “vanilla”	1	Sax	58.7	12.8	24	4.5
At 1:57 to 2:03 the piano does not pick up the turnaround	2	Commenting listener	54.1	17.4	24.4	4.1
The vamp ending was fun	3	Sax	48.8	20.7	26.0	4.5
The sax played a cliché ending at about 6:29	2	Pianist	44.6	14.0	40.9	0.4

Ratings of 4 and 5 on the 5-point scale (Agree and Strongly Agree) are collapsed into “Agree,” and Ratings of 1 and 2 (Strongly Disagree and Disagree) are collapsed into “Disagree.”

**Table 5:** **Percentages of listeners endorsing each of the 12 performance-specific statements that the performers had disagreed about, ranked from most to least endorsed**.

**Statement**	**About recording**	**Statement originator**	**Performer agreement**	**Agree (%)**	**Neither agree nor disagreed (%)**	**Disagree (%)**	**Don't under-stand (%)**
This version had the most motion	3	Sax	Disagreed (possibly not)	77.3	12.8	8.7	1.2
At 1:57 to 2:03 the sax plays a turnaround at the end of the melody to get back to the top of the sax solo	2	Commenting listener	Disagreed (ideological)	75.2	10.7	9.1	5.0
The pianist's opening was excellent	3	Sax	Disagreed	71.9	17.4	9.9	0.8
This version took the most harmonic liberties	3	Sax	Disagreed	71.5	14.5	9.9	4.1
During these two choruses starting at about 1:22 the sax hears and uses the pianist's substitutions	1	Commenting listener	Disagreed (possibly not)	69.8	16.9	7.9	5.4
At about 2:05 the players find the top together and are OK again	2	Commenting listener	Disagreed (ideological)	59.9	20.2	16.9	2.9
The pianist gave a cue to end at about 6:00 by using the pedal	1	Pianist	Disagreed (possibly not)	56.2	16.5	24.8	2.5
In the last phrase the sax played, the sax was “fishing” to get out of the tune	2	Sax	Disagreed	55.8	15.7	20.2	8.3
At 1:57 to 2:03 because the piano does not pick up the turnaround, things are a bit discombobulated between the two players	2	Commenting listener	Disagreed (ideological)	50.0	16.1	31.4	2.5
At about 4:52 the sax intended to play another chorus	3	Pianist	Disagreed (ideological)	39.7	26.9	31.0	2.5
When the pianist played in the same range as the sax at about 1:37, the pianist was stepping on the sax's toes	1	Pianist	Disagreed	33.9	12.4	50.4	3.3
When the pianist played a solo line over the sax from 1:53 to 1:59, the pianist was stepping on the sax's toes	1	Pianist	Disagreed	33.5	12.0	52.1	2.5

Ratings of 4 and 5 on the 5-point scale (Agree and Strongly Agree) are collapsed into “Agree,” and Ratings of 1 and 2 (Strongly Disagree and Disagree) are collapsed into “Disagree.”

Although some of the statements with the greatest endorsement seem to be more music-analytic than evaluative, and some statements with the least endorsement seem to be more evaluative (negative assessments) or to be judgments of performers' intentions, it is not clear that these kinds of distinctions explain the pattern of endorsements in a straightforward way. The overall pattern also isn't explained simply by “don't understand” ratings, which accounted for a relatively small proportion of the ratings, nor by listeners' unwillingness to commit to a judgment; for many statements more listeners explicitly disagreed than selected the “neither agree nor disagree” option.

To address Research Questions 1–3, we carried out a 2 × 2 × 2 mixed (within- and between-subjects) ANOVA on the ratings data, treating the ratings from 1 to 5 as continuous^3^, and treating ratings of “don't understand” as missing^4^. The within-subjects factor was whether the statements to be rated had been agreed about or disagreed about by the performers; the between-subjects factors were listener genre (whether listeners reported being jazz players or not) and listener instrument (whether they played the same instruments [saxophone and piano] as the performers) or not.

**Figure 3:**
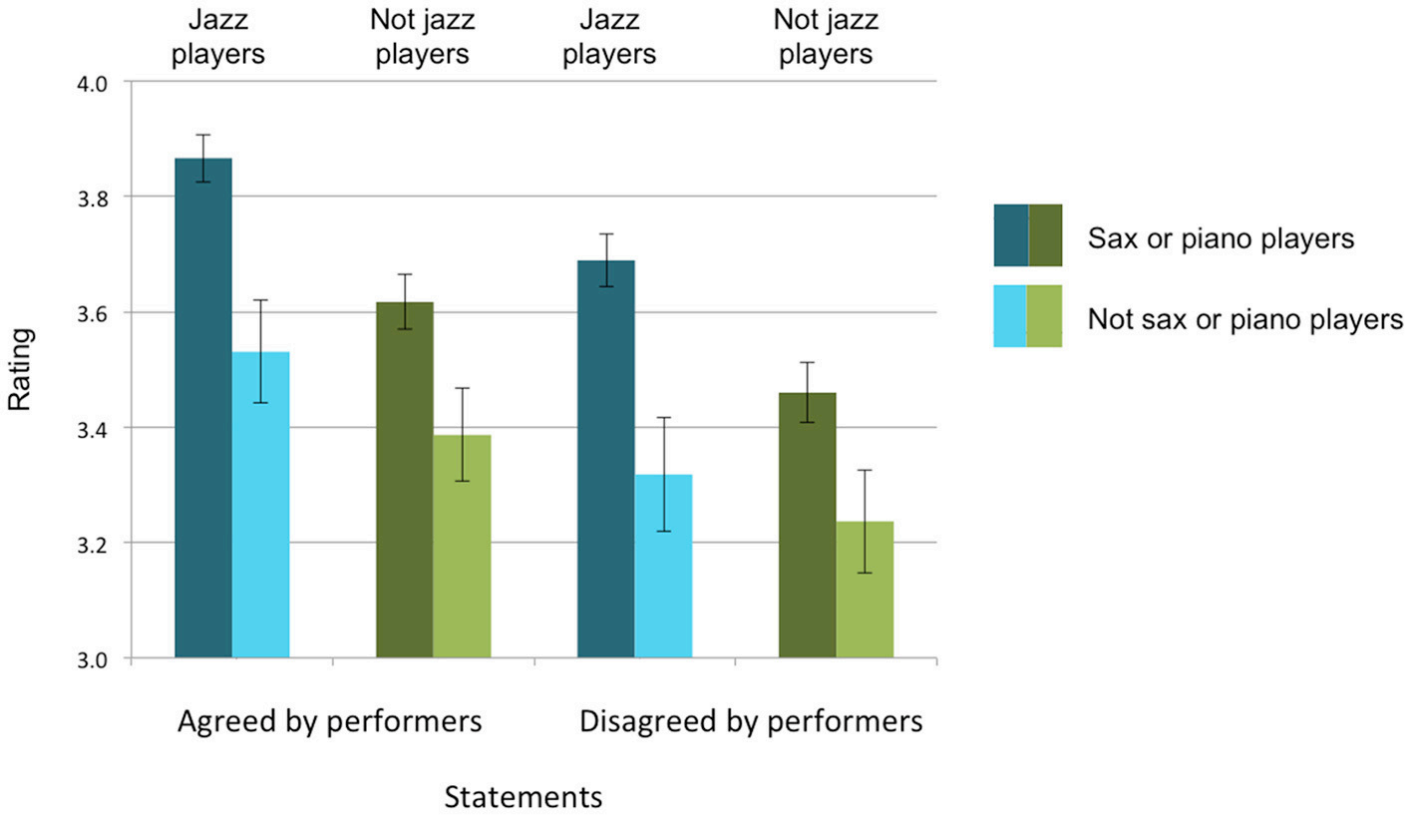
**Average levels of endorsement of statements**. All listeners were more likely to endorse statements that performers had agreed about (left half of figure) than statements performers had disagreed about (right half of figure). Listeners who classified themselves as jazz players endorsed statements more (blue bars) than non-jazz-players (green bars). Listeners who reported playing the same instruments as the performers (sax or piano) endorsed statements more (darker bars) than listeners who did not play sax or piano (lighter bars).

As Figure 3 shows, the answers to Research Questions 1–3 are all “yes,” with three independent main effects and no significant interactions. For Research Question 1 (performer agreement), despite the fact that the statements our listeners rated gave no indication of performers' levels of agreement about them, listeners endorsed statements that the performers had both endorsed more (3.60 on the 5-point scale) than statements the performers had disagreed about (3.43), *F*_(1, 235)_ = 30.537, *p* <0.001, η^2^ = 0.115 (a medium to large effect, see Cohen, 1988). For Research Question 2 (listener genre), listeners who were jazz players (*n* = 129) endorsed the statements more (3.60 on the 5-point scale) than listeners who were not jazz players (*n* = 110) (3.43 on the 5-point scale), *F*_(1, 235)_ = 7.550, *p* = 0.006, η^2^ = 0.031 (a small effect). For Research Question 3 (listener instrument), listeners who had played either sax or piano (*n* = 188) endorsed the statements (all of which had been made by sax or piano players) more (3.67 on the 5-point scale) than listeners who had not (*n* = 51) (3.37 on the 5-point scale), *F*_(1, 235)_ = 20.504, *p* <0.001, η^2^ = 0.080 (a medium to large effect). The findings for Research Question 2 and 3 were irrespective of whether the performers had agreed about the statements: there was no significant interaction with performer agreement.

Additional analyses demonstrate that our listeners were (collectively) quite sensitive in endorsing statements at levels that reflected performers' different kinds of agreement. In the Schober and Spiro (2014) analyses, subsequent re-interviews with the performers about the statements they disagreed about demonstrated that some of the disagreements might not be true disagreements (that is, they seem to have reflected different interpretations of terms in the statements), and others might have reflected ideological differences in how the performers were willing to talk about jazz as a genre, and thus might also not be true disagreements. In a second 4 × 2 × 2 ANOVA, we used the classification of the statements from Schober and Spiro (2014) as Agreed-upon, Possibly-Not-Disagreements, Ideological-Disagreements, and True Disagreements (see Table 5 for classifications of statements the performers disagreed about). In repeated contrasts, there was no difference in ratings for Agreed-upon statements and Possibly-Not-Disagreements, *F*_(1, 237)_ = 0.59, *p* = 0.444, η^2^ = 0.002; but significantly lower endorsement of the Ideological-Disagreements relative to the Possibly-Not-Disagreements, *F*_(1, 237)_ = 25.75, *p* <0.001, η^2^ = 0.098 (a medium to large effect), which did not differ reliably from the levels of endorsement of the True Disagreements relative to the Ideological-Disagreements, *F*_(1, 237)_ = 2.54, *p* = 0.112, η^2^ = 0.002. The other main effects of listener genre and listener instrument remained the same, and there were no significant interactions.

Taken together, the general pattern of endorsement ratings demonstrates that listeners' genre experience and instrumental experience do indeed affect their likelihood of agreeing with statements made by performers.^5^ Listeners are also less likely to endorse statements that the performers themselves truly disagreed about.

### Listeners' interrater agreement with performers and commenting listener

To address Research Question 4 (agreement with performers' vs. commenting listener's patterns of judgment), we used Cohen's kappa to compare interrater agreement between our listeners and the performers with the interrater agreement between our listeners and the commenting listener. We first calculated Cohen's kappas for each listener's (*n* = 239) ratings of the 24 specific statements with the sax player's and the pianist's ratings of those statements, collapsing the 5-point scale into three categories (Agree, Neutral, Disagree), and treating any ratings of “don't understand” as missing data. (The overall pattern is highly similar if we calculate kappas based on the 5-point scale). As one might expect given that we selected half the statements on the basis of the performers' having disagreed about them, the performers' own interrater agreement for these statements was extremely low, *K* = −0.279, and much lower than the performers' interrater agreement more generally (*K* = +0.280). We also calculated Cohen's kappas for the agreement of each listener's ratings with the commenting listener's ratings.

Given the large range in the resulting kappas (−0.339 to +0.600), we also generated a comparable data set with 239 instances of 24 randomly generated ratings from 1 to 3, to see what the range of kappas with this many ratings on a 3-point scale with this comparison set of performer and commenting listener ratings would look like; they ranged from −0.390 to +0.240. This allowed us to calibrate the extent to which the kappas for listener agreement with the performer and commenting listener ratings differed from chance. We take the highest value of the 239 random kappas with each performer and the commenting listener as our cutoff in order to set a high bar for assessing agreement—for judging that any particular listener agreed with a performer more than chance would predict^6^.

The large range of kappas we observe among the listeners shows that the performers' judgments are not uniformly shared across this listening audience, nor are the commenting listener's. A great number of listeners in this sample do not agree in their ratings with either player or the commenting listener more than chance would predict. As Table 6 shows, no listener agreed with the pianist's ratings beyond chance, under our conservative cutoff; far more listeners (68 of 239) gave ratings that agree beyond chance with the commenting listener's ratings.

**Table 6:** **Range of interrater agreement, using Cohen's kappas (κ), for listener (*n* = 239) and randomly generated (*n* = 239) ratings of the 24 specific statements compared with performers' and commenting listener's ratings**.

	**Minimum listener κ**	**Minimum random κ**	**Maximum listener κ**	**Maximum random κ**	**Number of listeners with κ > random**
Sax player	−0.339	−0.390	0.541	0.405	11
Pianist	−0.296	−0.226	0.318	0.400	0
Commenting listener	−0.316	−0.213	0.600	0.214	68

*Kappas are calculated collapsing the 5-point scale into three categories (Agree, Neutral, Disagree): ratings of 4 and 5 on the 5-point scale (Agree and Strongly Agree) are collapsed into “Agree,” and ratings of 1 and 2 (Strongly Disagree and Disagree) are collapsed into “Disagree.” Ratings of “don't understand” are treated as missing data*.

So the evidence from this dataset on Research Question 4 is consistent with one version of a *listeners-as-outsiders* view: more listeners agreed with another listener's judgments than with the performers' judgments.

To what extent does this evidence of low agreement with the performers reflect actual disagreement, as opposed to alternate interpretations of the wording in the statements, or ideological differences in talking about jazz? (Recall that some of the performers' disagreements may have resulted from different interpretations of wording or ideological differences). Although of course we cannot know for sure that our listeners' “disagree” ratings reflected true disagreement, we have some supporting evidence in opposing write-in comments by different listeners about the very same statements that suggest that, at least in those cases, the differences in numerical ratings reflected true disagreement with the content of the statements, rather than quibbles about the wording or ideological differences. For example, an endorser of the statement “At 1:57 to 2:03 because the piano does not pick up the turnaround, things are a bit discombobulated between the two players” wrote in “Sounds like they missed each other,” while a dissenter's comment (exact wording and spelling) was “its not jumbled in any way its rather fitting.” An endorser of “At about 6:10, the sax plays a classic wrap-up cliché” wrote in “Very Cliche, too obvious,” while a dissenter wrote “I don't think it was cliché it was a good mixture to it and signals the song was ending.” And an endorser of “At about 2:50 there was nice and memorable interplay” wrote “The two instruments clearly, beautifully, complement each other here,” while a dissenter wrote “timing was off.”

### Proximities between listeners', performers', and commenting listener's judgments

Even if many listeners' ratings of these statements did not line up with the performers' and commenting listener's ratings, were there any detectable patterns in the distribution of listeners' ratings? For Research Question 5, were ratings by listeners with jazz experience more similar to the performers' ratings than ratings by listeners without jazz experience? For Research Question 6, did listeners' instrumental experience lead them to make judgments more similar to the performers who played their instrument?

To address these questions, we approached the data in a different way, calculating a proximity matrix, using the SPSS hierarchical clustering routine (Ward's method), that represented the squared Euclidian distance between the pattern of 24 ratings for each listener and every other listener, as well as for the ratings by the performers and the commenting listener. Excluding the data for any listeners who gave any “don't understand” ratings, this left a dataset of 176 cases (173 listeners, the two performers, and the commenting listener), and a 176 × 176 proximity matrix, where smaller values indicate more similar ratings, and larger values indicate more dissimilar ratings.

**Figure 4:**
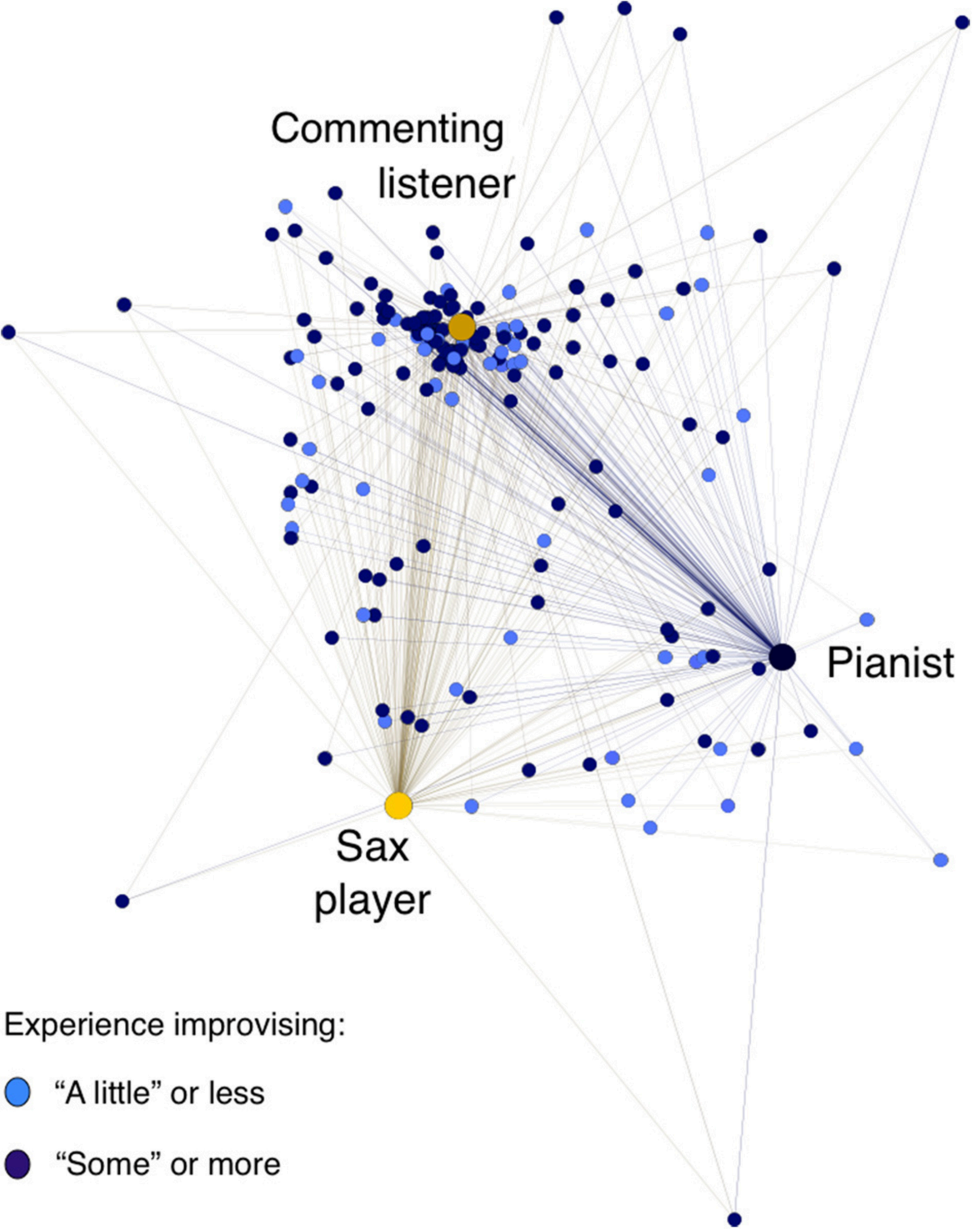
**Listeners' proximities across their 24 ratings with each other, the performers, and the commenting listener, distinguishing listeners with more and less experience improvising**. Raters with more similar ratings (a lower squared Euclidian distance) appear closer together, and raters with more dissimilar ratings (a higher squared Euclidian distance) appear farther apart. *This force-directed graph, representing the proximity matrix of each rater's squared Euclidian distance (across their 24 ratings) from every other rater's, was created using the ForceAtlas2 algorithm in Gephi 8.2, with setting of an approximate repulsion force of 1.2, a gravity force of 1.0, and a scaling of 2.0 (see Jacomy et al*., *2014)*.

Figure 4 represents this proximity matrix spatially in a force-directed graph that presents each rater's distance (across their 24 ratings) from every other rater's, using the ForceAtlas2 algorithm in the open-source Gephi 8.2 software (see Jacomy et al., 2014). In this representation, raters with more similar ratings (a lower squared Euclidian distance) appear closer together, and raters with more dissimilar ratings (a higher squared Euclidian distance) appear farther apart.

What is immediately apparent is that there was substantial variability in the listener sample: listeners could cluster in their judgments closer to the pianist, the saxophonist, or the commenting listener, or their judgments could be quite distant from everyone else's. No listener's judgments overlapped entirely with either performer's, and it was a small minority of listeners whose ratings grouped closely with either performer's.

Quantitatively, more listeners clustered closer to the commenting listener than to either performer. (This finding is thus consistent with the pattern evidenced by the Cohen's kappa analyses, supporting our Research Question 4 finding that more listeners were likely to agree with the commenting listener than with either performer.) As can be seen in Figure 4, listeners' average proximity score with the commenting listener (24.15) was significantly lower than their average proximity scores with both performers (32.24 with the pianist and 31.78 with the sax player), difference contrast *F*_(1, 173)_ = 178.98, *p* <0.0001, η^2^ = 0.508. Listeners' average proximity scores with the two players were not reliably different, difference contrast *F*_(1, 173)_ = 0.21, *p* = 0.645, η^2^ = 0.001.

To address Research Question 5, we asked whether listeners' genre expertise (measured in our questionnaire through the items about jazz listening and audience experience and jazz performing experience) led to greater agreement with performers' judgments, as measured by proximity ratings closer to the performers'. (Responses to the various different questions about jazz performing and listening experience were substantially intercorrelated, though not perfectly). By two measures we did see evidence that listeners with more jazz expertise did indeed have significantly lower proximity scores than listeners with less jazz expertise. First, the 125 listeners who reported having more experience improvising (“some” or more) gave ratings that were slightly closer to the performers' ratings (average proximity scores of 31.59 and 31.74 with the performers) and slightly farther from the commenting listener's ratings (average proximity score of 24.90) than the 49 listeners who reported less experience improvising (average proximity scores of 32.27 and 33.5 with the performers and 22.25 with the commenting listener), interaction contrast (listener genre expertise × performer vs. commenting listener) *F*_(1, 172)_ = 9.19, *p* = 0.003, η^2^ = 0.051. Second, the 72 listeners who reported listening to jazz more than 30 min per day gave ratings that were slightly closer to the pianist's ratings (average proximity score of 30.06) and slightly farther from the sax player's (average proximity score of (32.42) than the 101 listeners who reported listening to jazz less than 30 min per day (average proximity scores of 33.78 and 31.33, respectively), interaction contrast *F*_(1, 172)_ = 5.80, *p* = 0.017, η^2^ = 0.033. But in both cases these are minor effects (in terms of mean differences and effect sizes) relative to the overwhelming tendency for listeners to agree with the commenting listener more than the performers, as can be seen in Figure 4.

**Figure 5:**
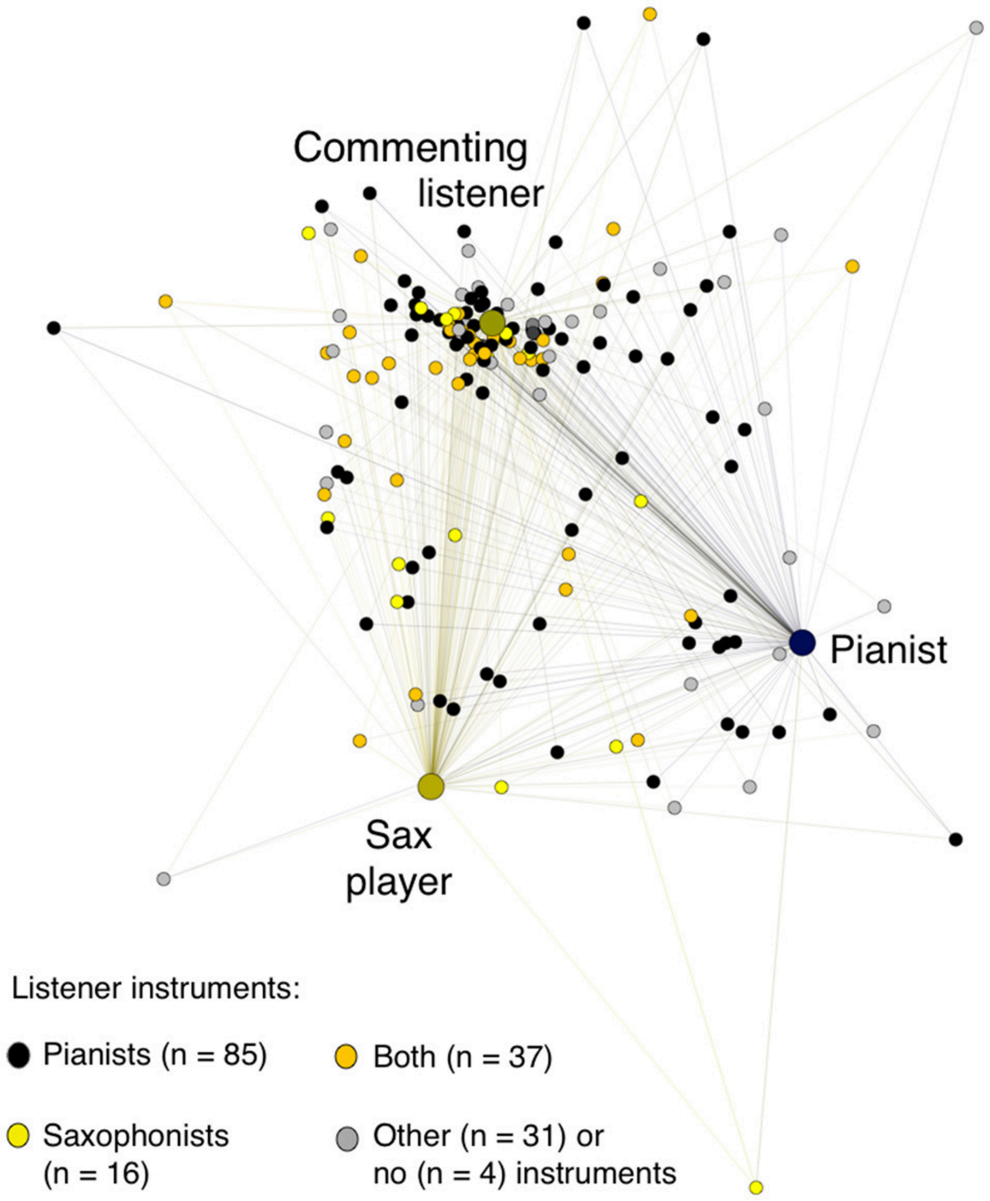
**Listeners' proximities across their 24 ratings with each other, the performers, and the commenting listener, distinguishing listeners by the instruments they reported playing**. Raters with more similar ratings (a lower squared Euclidian distance) appear closer together, and raters with more dissimilar ratings (a higher squared Euclidian distance) appear farther apart. *This force-directed graph, representing the proximity matrix of each rater's squared Euclidian distance (across their 24 ratings) from every other rater's, was created using the ForceAtlas2 algorithm in Gephi 8.2, with setting of an approximate repulsion force of 1.2, a gravity force of 1.0, and a scaling of 2.0 (see Jacomy et al.*, *2014**)*.

To address Research Question 6, we asked whether listeners with expertise playing the saxophone, piano, or both agreed more with judgments by the performers than listeners who played other instruments or no instrument. As Figure 5 shows, there was substantial variability: listeners whose pattern of ratings agreed most with the pianist's included some sax players, and the listeners whose pattern of ratings agreed most with the sax player included some pianists. And we see no quantitative evidence that pianist listeners' proximity scores were any closer to the pianist's than other instrumentalists', nor that sax players' proximity scores were any closer to the sax player's. (Note that our sample included many listeners who had experience on both instruments, which may make it harder to detect such effects if they exist). But we do see some quantitative evidence consistent with the idea that listeners' instrumental experience affected their listening: listeners who had experience playing *either* sax or piano or both had significantly lower proximity scores with the performers *and* the commenting listener (who was a sax player) (average proximity scores from 28.80 to 29.33) than did listener whose musical experience was with other instruments (average proximity score 32.28), difference contrast *F*_(1, 166)_ = 131.45, *p* <0.0001, η^2^ = 0.442.^7^

Exploratory analyses using the remaining questions about musical experience in our questionnaire uncovered no additional effects, with one exception: listeners with more years of musical practice (in any genre) were less likely to agree with (had higher proximity scores with) the pianist (32.82) than they did with the sax player (30.98), while people with fewer years of practice were more likely to agree with the pianist (33.56) and less likely to agree with the sax player (30.96), *F*_(1, 172)_ = 4.30, *p* = 0.040, η^2^ = 0.024. We are cautious about overinterpreting this effect, given the potential for finding spurious associations in multiple tests. In any case, in each comparison the overwhelming and statistically significant pattern was greater agreement with the commenting listener than either performer.

Looking more closely at the listeners whose ratings were outliers (particularly high proximity ratings), we did not see any notable pattern. Although a few listeners were outliers across all performances, many were outliers only once, and they did not have common musical experience (particular levels of experience or genre or instrumental backgrounds).

Our findings demonstrate that there was notable overlap among many individual listener judgments; while they *could* be idiosyncratic, there was a general tendency in judgment across the group. But the general tendency was, in this case, quite far from the performers', and much closer to the commenting listener's—even for listeners with greater relevant genre expertise. It clearly is possible for performers to have an interpretation of what happened that is shared with very few listeners.

## Discussion

These findings begin to quantify the range of interpretation across a listening audience, and demonstrate how different listeners' interpretations can be from performers'. They extend, to a different kind of interaction than has previously been studied, what is known about how participants in an interaction understand differently than non-participants: interpretations that performers themselves agree on are more likely to be shared with outsiders than interpretations they disagree on (Research Question 1). But the observed levels of agreement with the performers were low, with more listeners agreeing with the judgments of another commenting listener than with the performers (Research Question 4). Although we find some evidence that listeners' musical genre experience (Research Questions 2 and 5) and instrumental experience (Research Questions 3 and 6) affected their interpretations, there was still substantial variability: listeners with similar backgrounds could interpret the same performance quite differently.

How do these findings address the competing views of audience experience we laid out at the start? Our evidence is not consistent with a *radical idiosyncrasy* view: despite the range in judgments across 24 specific statements about the improvisations in our listener sample, at least some listeners' judgments grouped together. The fact that some listeners' judgments overlapped closely with other listeners' judgments is consistent with the *specific-content* view. The fact that different listeners could disagree about these very same statements is more consistent with the *minimal overlap* view that much less is shared. We found at least some evidence for the *more-experienced-listeners-understand-more-like-performers* view: “insider” listeners with jazz experience endorsed performers' statements more and had patterns of ratings that were slightly closer to the performers' (though this was a small effect), and listeners who had experience playing the same instruments the performers played agreed a bit more with the performers' ratings (a large effect). This suggests to us that the competing views on what is communicated in music may apply differently to listeners with different musical backgrounds: just as there may be audience sub-classes who differently share impressions of performer appropriateness (Platz and Kopiez, 2013), and just as people who have participated in, observed or simply heard about music therapy can have quite different judgments about the characteristics and effects of those musical interactions (Spiro et al., 2015), judgments of what is or is not communicated in a performance may vary across different sub-groups of listeners. And the extent to which these judgments overlap with performers' judgments may vary across different sub-groups of listeners.

Perhaps most strikingly, we saw clear evidence for the *listeners-as-outsiders* view: collectively listeners' ratings were far more likely to group with the commenting listener's judgments than with the performers' judgments. Across all our analyses, this comparison showed the largest differences, with a medium to large effect size.

### Measuring listeners' thinking

To our knowledge, this study measures listeners' understanding in a new way: asking them to rate their levels of agreement with specific statements about particular live performances, many of which had been generated independently by the performers themselves. As such, we see it as consistent with Radbourne et al.'s (2013a) call to develop new methods for gathering new kinds of data on audience experience. The focus was on listeners' responses to first encounters with performances, rather than on judgments further removed from the first moment of listening; on their judgments about a range of kinds of characterizations of music (rather than focused on affect or arousal); and on their responses to music chosen for them, rather than their responses to music they choose themselves.

We do not imagine that this method provides a complete account of listeners' perceptions and interpretations. Just as the performers' statements and ratings are not a perfect index of what the performers think—they may think other things we didn't ask about, some agreements may not be real agreements, and some disagreements may not be real disagreements—listeners are likely to have other reactions not tapped by our questionnaire. Different contexts of question-answering might have led to different reactions, just as they can in interviews with performers (Wilson and MacDonald, in press). And their ratings of our statements are, of course, filtered through their linguistic interpretations and ideological lenses; not all music listeners are linguistically sophisticated or linguistically able at all (e.g., Spiro and Himberg, 2016).

But the ratings do provide *one* index into listeners' thinking that gives some insight into listeners' shared understanding with the performers and each other. In our implementation, we intentionally included statements that we knew the performers disagreed about, so as to allow us to detect more clearly where listeners' judgments fell. In retrospect we can see that listeners ranged enormously in their endorsement even of statements the players had agreed about, so the benefit of our method of statement selection turned out to be in allowing us to see that listeners on average agreed more with statements the players had agreed about (whether because their ratings reflected listeners' implicit understanding of performers' shared interpretations or whether some statements simply *can* be endorsed more by anyone—performer or listener). In any case, the performance-specific statements chosen here seemed to be at the right level of detail so as to detect listener variability.

The fact that our method requires listeners to expend substantial time and effort in listening, comprehending statements, and making difficult judgments raises important questions about whether the method inherently leads to selection bias (attracting only the most dedicated and knowledgeable participants, and thus to samples of participants that do not reflect the general population of music listeners). We take some comfort from the evidence that our sample was not so demographically different from jazz audience members in the US (National Endowment for the Arts, 2009), and that the concert-going public may well be musically sophisticated (Pitts, 2013), but our recruitment method and the fact that so many of our listeners were trained musicians and music lovers do raise the questions of the extent to which this method allows generalization to the full complement of the jazz listening public, to listeners of other genres, or to listeners who are less focused and attentive to this survey task.

An additional methodological point: as the responses to our music experience questions demonstrated, listeners' musical experience doesn't always fall into neat categories, with musicians only having experience in one instrument or one genre. This is consistent with Finnegan's (1989) evidence that musical experience in a single community can be broader and more complex than is usually understood. The fact that we were able to observe some effects of listeners' genre and instrument on their judgments despite this suggests to us that effects of listeners' genre and instrument experience may be more powerful than our relatively small effect sizes suggest. In any case, it clearly is a challenge for audience research to understand how the multiple and overlapping dimensions of listeners' musical backgrounds contribute to their interpretations, and how best to recruit listeners with the characteristics of interest.

### Implications

How might these findings generalize to other performers or performances, other audiences and listening contexts, or other genres of music-making?

We see our findings as demonstrating what *can* happen rather than what is guaranteed always to happen. Our study examined listeners' judgments about characterizations by one pair of improvisers on a jazz standard, who were playing together for the first time, and by one commenting listener with genre expertise (as opposed to multiple listeners, or non-expert commenting listeners). It required focused listening and judgment by solo listeners who were not copresent with the performers and who could not see video of the performers, and so additional factors that can affect audience experience (e.g., eye contact between performers and audience, performers' gestures, performers' appearance) were not at play. It required listening in a situation without other audience members present, and with no additional evidence about what other listeners thought.

How exactly these findings generalize to other performers, listeners, listening contexts—which continue to expand (Clarke, 2005; Pitts, 2005, 2016; Pitts and Spencer, 2008; Schober, 2014)—and audience measures remains to be seen. The range of possible combinations of features is enormous, but from existing evidence and our own experience we hypothesize that the following variables (at least) could plausibly affect how likely listeners are to share understanding with performers and with each other:

- *Performer characteristics (individual and joint)*: experience as musicians; experience playing *this* style or *this* piece; experience playing with each other; overlap with listeners' cultural backgrounds or demographic characteristics.
- *Music characteristics*: genre; degree of improvisation or scriptedness; virtuosic demand; collaborative challenge; number of performers.
- *Music-making situation:* intended for an audience or not (performance vs. rehearsal); large or small or no audience; live vs. recorded; once only vs. multiple takes; able to see each other or not; able to influence and react to other performers vs. playing with recorded track.
- *Listener musical expertise:* playing experience (in this or other genres), instrument experience (on performer instruments or others), musical training, prior listening experience (in this or other genres), prior knowledge of the piece.
- *Other listener characteristics:* attentiveness; ability to reflect on musical experiences; patience and motivation for providing ratings in a study; overlap with performers' and other listeners' cultural backgrounds or demographic characteristics (e.g., age or generation); cognitive styles; perspective-taking ability or empathy.
- *Listening situation:* live vs. recorded performance; co-present with performers or not; listening alone or with others; having evidence of other listeners' reactions (beforehand, during, or after listening); extent to which listeners chose performers, piece, venue, etc.; degree of focal attention; whether listeners can or do relisten.
- *Kind of interpretation*: music-analytic characteristics; judgments of performers' intentions; emotional responses; characterization of performers' actions.

If the findings from the current study generalize, then more listeners should agree with a commenting listener's interpretations than with performers' interpretations even when performers have different characteristics than those in this study, when the music has different characteristics, in different music-making situations; and this should be the case even with listeners with different characteristics in different listening situations, and with regards to a range of different kinds of interpretations. Similarly, listeners with more genre experience should agree more with performers across all these different variables. But of course testing this would require new comparisons for all these different features.

For some features in this list, existing evidence suggests directional hypotheses about their effects on listeners' shared understanding with performers or other listeners. For example, physically copresent audience members in non-musical contexts can be affected by the reactions of those around them (see e.g., Pennebaker, 1980; Mann et al., 2013), and listeners' evaluations of the quality of music in an online marketplace can be affected by their knowledge of the evaluative judgments of other listeners (Salganik et al., 2006). This suggests that listeners who are aware of other listeners' reactions should end up with more similar interpretations to each other, although it doesn't suggest how similar their interpretations might be with performers'. As another example, the fact that musicians who scored on the “innovative” end of a cognitive styles measure generated more ideas in a concept mapping task than “adaptors” (Stoyanov et al., 2016) suggests that perhaps listeners who share this cognitive style with a performer or other listeners will be more likely to share understanding of a performance.

For other features in the list, existing evidence demonstrates effects on audience members, but it is less clear whether those effects generalize to listeners' shared understanding with performers or with each other. For example, the facts that eye contact between performers and audience members (e.g., Antonietti et al., 2009) or visual characteristics of the performers (Davidson, 2001, 2012; Thompson et al., 2005; Mitchell and MacDonald, 2012; Morrison et al., 2014) can affect audience members' judgments do not clearly point to whether audience members' interpretations will therefore be more similar to each other as a result, or more similar to performers'. Similarly, the fact that audience members can react differently to comparable live vs. recorded performances (see Barker, 2013; Katevas et al., 2015) doesn't clearly predict in which situation they are more likely to share understanding with performers or each other.

We see our findings, which start from what the performers themselves thought about the improvisations, as complementing findings from studies that measure other aspects of listening experience, such as listeners' physiological responses (e.g., Bachrach et al., 2015; Fancourt and Williamon, 2016), their judgments of the expressiveness of music performances (e.g., chapters in Fabian et al., 2014), or their continuous ratings of emotions in the music (Timmers et al., 2006; Schubert, 2011). Because listeners' judgments in our study range across the kinds of topics that the performers thought worth commenting on, they give insight about listener-performer shared understanding in a broad way that we see as reflecting the broad range of potential overlap and non-overlap. But they do not give systematic evidence on listener-performer or listener-listener overlap in more focused aspects of the listening experience (for an example of such a focused exploration, see Canonne and Garnier, 2015 on the extent to which listeners' segmentation of free jazz improvisations corresponds with performers'). We see this as an area ripe for further investigation.

More broadly, we see a connection between our findings on audience interpretation in music-making and questions about participants' and observers' interpretations of joint actions more generally. As collaborative views of joint action (e.g., Clark and Wilkes-Gibbs, 1986; Clark, 1996) note, what a participant or performer in a joint action intends isn't necessarily the same as what is understood by their collaborating partner, nor necessarily the same as what is understood by a non-participating observer or audience member (Schober and Clark, 1989; Wilkes-Gibbs and Clark, 1992). Based on our findings here, it makes sense to predict that observers of joint actions in other domains where the joint action could be a public display—dancing, conversing, even shaking hands—should be more likely to agree with an outsider's or each other's interpretations than with the participants', and that observers who have more experience in a domain are more likely to share understanding with the participants.

Despite how much is unknown about listener-performer shared understanding, our findings demonstrate that listeners' interpretations of what happened in a musical performance can be quite different from performers' interpretations, at least for this audience and these performances. Listeners' genre and instrumental expertise can affect their interpretations, but the strongest evidence supports the *listeners-as-outsiders* hypothesis: more listeners agree with an outsider's interpretations than with the performers'.

## Author contributions

Both authors contributed extensively to the design, data collection, and analyses presented here, as well as authoring this paper.

### Conflict of interest statement

The authors declare that the research was conducted in the absence of any commercial or financial relationships that could be construed as a potential conflict of interest.

## Footnotes

^1^This implementation was not optimized for use on mobile devices, which would have required a quite different presentation strategy given the complexity of the screen images.

^2^Unfortunately the Qualtrics platform did not allow us to log listeners' clicks or relistening within a screen of the survey, which might be useful indicators of listeners' conscientiousness or level of familiarity with the recordings. The time logs to which we have access are thus ambiguous about whether they reflect relistening, time thinking, or even inattentiveness.

^3^Using parametric statistical analyses on these rating scale data is supported by Norman's (2010) arguments and evidence about the robustness of parametric analyses for interval data. This does not, of course, mean that we are claiming that our listeners treated the distances between intervals on our scales as equal, which is unknowable (Jamieson, 2004); we simply are claiming that higher ratings are higher.

^4^The aggregated rating for each listener of the 12 statements the performers had agreed about and the 12 statements the performers had disagreed about omitted the “don't understand” ratings, which were rare. All listeners therefore had usable ratings of both kinds.

^5^Whether the finding on genre experience reflects generalized jazz knowledge or experience with “It Could Happen to You” is unknowable given that our musical experience questionnaire didn't ask directly about familiarity with the piece. But it is plausible that most experienced jazz players and listeners have familiarity with the piece.

^6^This procedure was suggested by one of the reviewers of Schober and Spiro (2014), Patrick Healey.

^7^So few listeners in this reduced dataset of 167 cases had no instrumental experience that we did not include them in this analysis.
