# Supplementary material for: Listeners' and Performers' Shared Understanding of Jazz Improvisations
Source: Front Psychol. 2016 Nov 2;7:1629. doi: 10.3389/fpsyg.2016.01629 (PMC5089999; doi:10.3389/fpsyg.2016.01629)
Supplement: Supplementary file 1 [file Table1.PDF]

SUPPLEMENTAL DATA: TABLE 1
COMPLETE ONLINE SURVEY

Q1.1 Welcome to this study! We hope you'll find it enjoyable and interesting.

We are interested in how you hear and interpret music, whether you're a performing musician yourself, a frequent audience member, or someone who hardly listens to music at all.

You will be asked to listen carefully to recordings of three live performances by a pair of professional musicians and rate how much you agree or disagree with statements about those performances. This will require a real investment of your time—at least an hour, possibly more—and you will need to be in a place where you can listen attentively without being interrupted. You will also need to be using a computer with an internet connection that allows you to buffer YouTube videos. In exchange you will receive a $20 Amazon.com gift code for completing the study.

We are really interested in what you hear and how you think about what you are hearing, whether or not you are able to use or understand technical musical vocabulary. This isn't a test with right or wrong answers! It's about your opinions and reactions, including hearing from you that you DON'T understand something.

Doing this will require listening to the music seriously, and probably more than once. If you aren't prepared to take the time to do this, this isn't the study for you.

Please continue to find out if you are eligible to participate.

Q2.1 Do you consider yourself, or have you ever considered yourself, a performing musician?
- ❍ Yes
- ❍ No

Answer If Do you consider yourself a performing musician? Yes Is Selected
Q2.2 What type of music do you (or did you) primarily perform?
- ❍ Jazz
- ❍ Classical
- ❍ Folk
- ❍ Rock
- ❍ Pop
- ❍ Something else ____________________

Answer If Do you consider yourself a performing musician? Yes Is Selected

Q2.3 What kind of instrument do you (or did you) primarily play?

- ❍ Keyboard (e.g., piano, organ, harpsichord, electronic keyboard)
- ❍ Wind instrument (e.g., saxophone, flute, oboe)
- ❍ Brass instrument (e.g. trumpet, trombone, horn)
- ❍ String instrument (e.g., violin, guitar, cello, sitar)
- ❍ Percussion (e.g., drums, xylophone)
- ❍ Voice/singing
- ❍ Something else ____________________

Answer If What is your primary instrument? GROUP - SINGLE ANSWER? keyboard (e.g., piano, organ, harpsichord) Is Selected

Q2.4 What keyboard instrument is (or was) your primary instrument?

- ❍ Piano
- ❍ Organ
- ❍ Harpsichord
- ❍ Electronic keyboard
- ❍ Harmonium
- ❍ Accordion
- ❍ Other ____________________

Answer If What is your primary instrument? GROUP - SINGLE ANSWER? wind instrument (e.g., saxophone, flute, trumpet) Is Selected

Q2.5 Which wind instrument is (or was) your primary instrument?

- ❍ Saxophone
- ❍ Clarinet
- ❍ Oboe
- ❍ Bassoon
- ❍ Flute
- ❍ Piccolo
- ❍ Shakuhachi
- ❍ Harmonica
- ❍ Didgeridoo
- ❍ Other ____________________

Answer If What kind of instrument do you primarily play? Brass instrument (e.g. trumpet, trombone, horn) Is Selected

Q2.6 Which brass instrument is (or was) your primary instrument?

- ❍ Trumpet
- ❍ French Horn
- ❍ Tuba
- ❍ Trombone
- ❍ Other ____________________

Answer If What kind of instrument do you primarily play? String instrument (e.g., violin, guitar, cello, sitar) Is Selected

Q2.7 Which string instrument is (or was) your primary instrument?

- Guitar (acoustic or electric)
- Bass guitar
- Violin
- Viola
- Cello
- Double Bass
- Banjo
- Ukulele
- Sitar
- Harp
- Kora
- Sarod
- Erhu
- Koto
- Oud
- Bazouki
- Uhadi
- Santar
- Other ____________________
-

Answer If What kind of instrument do you primarily play? Percussion (e.g., drums, xylophone) Is Selected

Q2.8 Which percussion instrument is (or was) your primary instrument?

- Drums
- Orchestral percussion instruments
- Xylophone
- Marimba
- Mbira
- Tabla
- Steel pan
- Other ____________________

Q2.9 What is your current age?

- Less than 18
- 18 to 24
- 25 to 34
- 35 to 44
- 45 to 54
- 55 to 64
- 65 or over

Q3.1 Thanks! You are eligible to participate in this study. Please read the following text and click "I Agree" if you consent to participate in this study and upon completion to provide your email address to receive the $20 electronic Amazon.com gift code.

The Study

In this study, you will be asked to listen carefully to three performances of a jazz standard and to rate the extent to which you agree with statements about these performances. You will also answer some questions about yourself. The study lasts approximately one hour; you should be in a place where you can listen attentively

without interruption. There are no anticipated risks or discomforts associated with this research. Through this work, we hope to gain a better understanding of how people hear and understand music.

It is important to point out that this is basic research on music listening. We do not evaluate individual responses. Rather, we are interested in general trends. Individual results are confidential. Research participants are identified only by an assigned ID number, with no names of participants connected with responses. Consent Agreement
I have read and understood the above statement and voluntarily consent to participate in this study, which is being carried out by Michael Schober and Neta Spiro at the New School for Social Research in New York City and the Research Department at Nordoff Robbins, London. I understand that I may refuse to participate before the study begins, discontinue at any time, or skip any questions without penalty. I understand that I will only receive the $20 electronic Amazon.com gift code if I complete the online questionnaire. Further, I consent to the publication of study results as long as the information is anonymous and disguised so that I cannot be identified. I also understand that concerns about any aspect of this project may be reported to the principal investigators at musicstudy@newschool.edu or at +1 (212) 229-5727 (Michael Schober) or +44 (0)20 7267 4496 (Neta Spiro). I may also contact—anonymously, if desired—the New School Institutional Review Board Human Subjects Committee coordinator, at (212) 229- 5727 ext. 3102.

I have understood the above explanation and give consent to my voluntary participation in this research project.
❍ I agree
❍ No thanks—I do not agree

Q4.1 Thanks—let’s get started.

You will be listening to three different recordings of “It Could Happen to You” by the same two performers, a pianist and a saxophonist. The performers are both professional musicians who had never met; they played in a performance space without being able to see each other, and their task was to create three very different versions together without talking about what they intended to do.

We need you to listen closely and attentively to each performance, and then to answer questions about your impressions. Some of the questions will be quite specific about what happened at particular moments; you will have a chance to listen again, as often as you like, to all or parts of each performance as you answer questions.

For each question, all we need is for you to give us a rating, but there is also a space in case you’d like to add any (optional) comment.

Q4.2 If you are not in a place now where you can listen without interruption but you will be able to listen on this device in this browser at a different time, you can rejoin the study when it's convenient by clicking on the link that you used to join the study.

If you won't be able to use this device and this browser to continue, please start from the beginning on a device that you can use for the study. (You will need an internet connection that allows you to buffer YouTube videos). If you need to take a break, you can keep the browser window open; if you close the browser window you can rejoin by clicking on the link that you used to join the study.

Q4.4 Recording 1
Please listen to Recording 1 in its entirety, without interruption, at least once. (You can play it more than once if you like). Press play when you are ready to begin. The button for proceeding to the next question will appear at the end of the recording.

If you have any problems with playing the recording, try waiting until the pale gray bar shows that the file is fully loaded before pressing play. When you have finished, continue to the next page to start answering questions about this recording.

Q4.5 Recording 1
Please rate the extent to which you agree or disagree with the following statements. The statements that you'll be rating range from the more subjective to the more technical. If you're not a musician or a jazz expert, you might find the technical ones difficult, but don't worry—we're not interested in "right" or "wrong" answers; we really want to know what you felt you heard. Please do use your personal judgement when rating the more subjective statements—we are genuinely interested in your opinion. If you do not understand a statement, please click "DON'T UNDERSTAND" rather than rating the statement. We are just as interested in whether the statements make sense to you as in any rating. If you have the time, we would be interested to know (in the optional "Comments" section) what exactly you are not understanding. You can listen to the recording again as many times as you wish - click the play button to begin the playback and scroll back and forth as many times as necessary.

	Strongly Disagree (1)	Disagree (2)	Neither Agree nor Disagree (3)	Agree (4)	Strongly Agree (5)	DON'T UNDERSTAND (6)
The overall performance was standard or “vanilla.”	❍	❍	❍	❍	❍	❍

Q4.6 Any comments? (optional)

Q4.7 Recording 1 - From about 1:22

These questions concern the music that starts at about 1:22; the file is already preset to start there (as you will see in the timer information at the bottom of the Recording 1 window). Just press the play button and the recording will start at 1:22.

Please do listen again before answering; you can also start from earlier, and listen again as often as you like. It may be useful to look at the questions first to know what you are listening for.

	Strongly Disagree (1)	Disagree (2)	Neither Agree nor Disagree	Agree (4)	Strongly Agree (5)	DON'T UNDERSTAND (6)
(a) Starting at about 1:22, the sax takes two choruses*. *chorus: a part of a song that recurs at intervals, usually following each verse; refrain. (from Dictionary.com)	❍	❍	❍	❍	❍	❍
(b) During these two choruses starting at about 1:22 the sax hears and uses the pianist's substitutions* *Chord substitution is the technique of using a chord in the place of another, often related, chord in a chord progression. Jazz musicians often substitute chords in the original progression to create variety and add interest to a piece. (from Wikipedia)	❍	❍	❍	❍	❍	❍
(c) When the pianist played in the same range* as the sax at about 1:37, the pianist was stepping on the sax's toes *The same general span of notes (same general pitch height)	❍	❍	❍	❍	❍	❍
(d) When the pianist played a solo line over the sax from 1:53 to 1:59, the pianist was stepping on the sax's toes.	❍	❍	❍	❍	❍	❍

Q4.8 Any comments about any of these statements (a, b, c, or d)? (optional)

- ❑ (a) ______________________
- ❑ (b) ______________________
- ❑ (c) ______________________
- ❑ (d) ______________________

Q4.9 Recording 1 - From about 4:39

This question concerns the music at about 4:39. The music is preset to start earlier (late in the sax solo, and before the piano solo starts), but you can listen wherever you like. Please do listen to the music again before you answer the question.

	Strongly Disagree (1)	Disagree (2)	Neither Agree nor Disagree (3)	Agree (4)	Strongly Agree (5)	DON'T UNDERSTAND (6)
At about 4:39 end of piano solo, the pianist played the same chord that the sax played at the end of the sax solo.	❍	❍	❍	❍	❍	❍

Q4.10 Any comments? (optional)

Q4.11 Recording 1 - From about 6:00

This question concerns the music at 6:00. The music is set to start a little before that.

	Strongly Disagree (1)	Disagree (2)	Neither Agree nor Disagree (3)	Agree (4)	Strongly Agree (5)	DON'T UNDERSTAND (6)
(a) The pianist gave a cue to end at about 6:00 by using the pedal.	❍	❍	❍	❍	❍	❍
(b) At about 6:10, the sax plays a classic wrap-up cliché.	❍	❍	❍	❍	❍	❍

Q4.12 Any comments about either of these statements (a or b)? (optional)

- ❑ (a) ______________________
- ❑ (b) ______________________

Q4.14 Recording 2

Please listen to Recording 2 in its entirety, without interruption, at least once. (You can play it more than once if you like). Press play when you are ready to begin. The button for proceeding to the next question will appear at the end of the recording. When you have finished, continue to the next page to start answering questions about this recording.

Q4.15 Recording 2 - From about 1:57

These questions concern the music from about 1:57. The music starts a little before that.

	Strongly Disagree (1)	Disagree (2)	Neither Agree nor Disagree (3)	Agree (4)	Strongly Agree (5)	DON'T UNDERSTAND (6)
(a) At 1:57 to 2:03 the sax plays a turnaround* at the end of the melody to get back to the top of the sax solo. *In jazz, a turnaround is a passage at the end of a section which leads to the next section. This next section is most often the repetition of the previous section or the entire piece or song (from Wikipedia)	❍	❍	❍	❍	❍	❍
(b) At 1:57 to 2:03 the piano does not pick up the turnaround.	❍	❍	❍	❍	❍	❍
(c) At 1:57 to 2:03 because the piano does not pick up the turnaround, things are a bit discombobulated between the two players.	❍	❍	❍	❍	❍	❍
(d) At about 2:05 the players find the top* together and are OK again. *The beginning point of each chorus, the first beat of the first measure (definition by Darius Brotman)	❍	❍	❍	❍	❍	❍

Q4.16 Any comments about any of these statements (a, b, c, or d)? (optional)

❑ (a) ______________________
❑ (b) ______________________
❑ (c) ______________________
❑ (d) ______________________

Q4.17 Recording 2 - from around 2:40

These questions concern the music from about 2:40. The music starts a little before that.

	Strongly Disagree (1)	Disagree (2)	Neither Agree nor Disagree (3)	Agree (4)	Strongly Agree (5)	DON'T UNDERSTAND (6)
(a) From about 2:40, the sax signaled the end of one chorus and the beginning of the next.	❍	❍	❍	❍	❍	❍
(b) At about 2.50 there was nice and memorable interplay.	❍	❍	❍	❍	❍	❍
(c) The pianist continued the sax's phrasing at about 2:55.	❍	❍	❍	❍	❍	❍

Q4.18 Any comments about any of these statements (a, b, or c)? (optional)

❑ (a) ______________________
❑ (b) ______________________
❑ (c) ______________________

Q4.19 Recording 2 - from around 6:00 to the end

These questions concern the music from about 6:00. The music starts a little before that.

	Strongly Disagree (1)	Disagre e (2)	Neither Agree nor Disagree (3)	Agree (4)	Strongly Agree (5)	DON'T UNDERSTAND (6)
(a) In the last phrase the sax played, the sax was "fishing" to get out of the tune.	❍	❍	❍	❍	❍	❍
(b) The sax played a cliché ending at about 6:29.	❍	❍	❍	❍	❍	❍

Q4.20 Any comments about either of these statements (a or b)? (optional)

❑ (a) ______________________
❑ (b) ______________________

Q4.22 Recording 3

Please listen to Recording 3 in its entirety, without interruption, at least once. (You can play it more than once if you like). Press play when you are ready to begin. The button for proceeding to the next question will appear at the end of the recording. When you have finished, continue to the next page to start answering questions about this recording.

Q4.23 Recording 3

These questions concern Recording 3 as a whole.

(Just in case you want them for these two questions, Recordings 1 and 2 are also reposted below, but listening to them is optional).

	Strongly Disagree (1)	Disagree (2)	Neither Agree nor Disagree (3)	Agree (4)	Strongly Agree (5)	DON'T UNDERSTAND (6)
(a) This version took the most harmonic liberties.	❍	❍	❍	❍	❍	❍
(b) This version had the most motion.	❍	❍	❍	❍	❍	❍

Q4.24 Any comments about either of these statements (a or b)? (optional)

❑ (a) ____________________

❑ (b) ____________________

Q4.26 Recording 3 - The beginning

These questions concern the beginning of Recording 3.

	Strongly Disagree (1)	Disagree (2)	Neither Agree nor Disagree (3)	Agree (4)	Strongly Agree (5)	DON'T UNDERSTAND (6)
(a) The pianist set the tempo.	❍	❍	❍	❍	❍	❍
(b) The pianist's opening was excellent.	❍	❍	❍	❍	❍	❍

Q4.27 Any comments about either of these statements (a or b)? (optional)

❑ (a) ____________________

❑ (b) ____________________

Q4.28 Recording 3 - From about 1:38
These questions concern the music from about 1:38.

	Strongly Disagree (1)	Disagree (2)	Neither Agree nor Disagree (3)	Agree (4)	Strongly Agree (5)	DON'T UNDERSTAND (6)
(a) At about 1:38 the sax begins the sax's second chorus and the piano begins accompanying it.	❍	❍	❍	❍	❍	❍
(b) During this chorus, the sax also plays a somewhat fragmented improv, and with lines that pull away from the harmony.	❍	❍	❍	❍	❍	❍

Q4.29 Any comments about either of these statements (a or b)? (optional)
❑ (a) ____________________
❑ (b) ____________________

Q4.30 Recording 3 - From about 4:52
This question concerns the music from about 4:52. The music starts a little before that.

	Strongly Disagree (1)	Disagree (2)	Neither Agree nor Disagree (3)	Agree (4)	Strongly Agree (5)	DON'T UNDERSTAND (6)
At about 4:52 the sax intended to play another chorus.	❍	❍	❍	❍	❍	❍

Q4.31 Any comments? (optional)

Q4.32 Recording 3 - From about 5:04
This question concerns the music from about 5:04.

	Strongly Disagree	Disagree (2)	Neither Agree nor Disagree (3)	Agree (4)	Strongly Agree (5)	DON'T UNDERSTAND (6)
The vamp* ending was fun.<br>*A simple section like a riff, designed to be repeated as often as necessary (definition by Darius Brotman)	❍	❍	❍	❍	❍	❍

Q4.33 Any comments? (optional)

Q5.1 Great! You've gotten through the hardest part. This next part should be quicker and easier; we want to know your general impressions of the three recordings relative to each other.   Here you will be asked a new set of questions about each of the three recordings – the same set of questions about each recording.

You will be able to listen to each recording again if you like, as many times as you like, while you answer the questions. As before, if you don't understand a statement, please pick "Don't understand"; this isn't a test with right or wrong answers.

If you want to change your answers along the way, you will be able to go back and do so.

Just as before, for each question, all we need is for you to give us a rating, but there is also a space at the end of each page in case you'd like to add any (optional) comments.

Q5.2 Recording 1 In my opinion:

	Strongly Disagree (1)	Disagree (2)	Neither Agree nor Disagree (3)	Agree (4)	Strongly Agree (5)	DON'T UNDERSTAND (6)
This performance was artistically successful.	❍	❍	❍	❍	❍	❍
The ensemble sounded unified.	❍	❍	❍	❍	❍	❍
The interplay between piano and sax was excellent.	❍	❍	❍	❍	❍	❍
The balance in dynamics (volume) between piano and sax was excellent.	❍	❍	❍	❍	❍	❍
The rhythmic coordination between piano and sax was excellent.	❍	❍	❍	❍	❍	❍
The pianist's improvisation fit what the sax was doing.	❍	❍	❍	❍	❍	❍
The sax's improvisation fit what the pianist was doing.	❍	❍	❍	❍	❍	❍
The pianist's improvisation was coherent.	❍	❍	❍	❍	❍	❍
The sax's improvisation was coherent.	❍	❍	❍	❍	❍	❍
The pianist's improvisation was creative.	❍	❍	❍	❍	❍	❍
The sax's improvisation was creative.	❍	❍	❍	❍	❍	❍
The pianist's playing was excellent.	❍	❍	❍	❍	❍	❍
The sax's playing was excellent.	❍	❍	❍	❍	❍	❍
The overall performance was excellent.	❍	❍	❍	❍	❍	❍

Q5.3 Any comments? (optional)

Q5.4 Recording 2 In my opinion:

	Strongly Disagree (1)	Disagree (2)	Neither Agree nor Disagree (3)	Agree (4)	Strongly Agree (5)	DON'T UNDERSTAND (6)
This performance was artistically successful.	❍	❍	❍	❍	❍	❍
The ensemble sounded unified.	❍	❍	❍	❍	❍	❍
The interplay between piano and sax was excellent.	❍	❍	❍	❍	❍	❍
The balance in dynamics (volume) between piano and sax was excellent.	❍	❍	❍	❍	❍	❍
The rhythmic coordination between piano and sax was excellent.	❍	❍	❍	❍	❍	❍
The pianist's improvisation fit what the sax was doing.	❍	❍	❍	❍	❍	❍
The sax's improvisation fit what the pianist was doing.	❍	❍	❍	❍	❍	❍
The pianist's improvisation was coherent.	❍	❍	❍	❍	❍	❍
The sax's improvisation was coherent.	❍	❍	❍	❍	❍	❍
The pianist's improvisation was creative.	❍	❍	❍	❍	❍	❍
The sax's improvisation was creative.	❍	❍	❍	❍	❍	❍
The pianist's playing was excellent.	❍	❍	❍	❍	❍	❍
The sax's playing was excellent.	❍	❍	❍	❍	❍	❍
The overall performance was excellent.	❍	❍	❍	❍	❍	❍

Q5.5 Any comments? (optional)

Q5.6 Recording 3 In my opinion:

	Strongly Disagree (1)	Disagree (2)	Neither Agree nor Disagree (3)	Agree (4)	Strongly Agree (5)	DON'T UNDERSTAND (6)
This performance was artistically successful.	❍	❍	❍	❍	❍	❍
The ensemble sounded unified.	❍	❍	❍	❍	❍	❍
The interplay between piano and sax was excellent.	❍	❍	❍	❍	❍	❍
The balance in dynamics (volume) between piano and sax was excellent.	❍	❍	❍	❍	❍	❍
The rhythmic coordination between piano and sax was excellent.	❍	❍	❍	❍	❍	❍
The pianist's improvisation fit what the sax was doing.	❍	❍	❍	❍	❍	❍
The sax's improvisation fit what the pianist was doing.	❍	❍	❍	❍	❍	❍
The pianist's improvisation was coherent.	❍	❍	❍	❍	❍	❍
The sax's improvisation was coherent.	❍	❍	❍	❍	❍	❍
The pianist's improvisation was creative.	❍	❍	❍	❍	❍	❍
The sax's improvisation was creative.	❍	❍	❍	❍	❍	❍
The pianist's playing was excellent.	❍	❍	❍	❍	❍	❍
The sax's playing was excellent.	❍	❍	❍	❍	❍	❍
The overall performance was excellent.	❍	❍	❍	❍	❍	❍

Q5.7 Any comments? (optional)

Q6.1 Great! You're finished with the questions that require listening to the recordings. Now we have just a few questions about the performers and about you. These are about your overall impressions of the players. In my opinion:

	Strongly Disagree (1)	Disagree (2)	Neither Agree nor Disagree (3)	Agree (4)	Strongly Agree (5)	DON'T UNDERSTAND (6)
Both players listen to a lot of the same stuff.	❍	❍	❍	❍	❍	❍
The sax is open to doing whatever happens in the moment.	❍	❍	❍	❍	❍	❍
The pianist is open to doing whatever happens in the moment.	❍	❍	❍	❍	❍	❍
The sax is well-rounded as a musician.	❍	❍	❍	❍	❍	❍
The pianist is well-rounded as a musician.	❍	❍	❍	❍	❍	❍
The sax's signals are very clear.	❍	❍	❍	❍	❍	❍
The pianist's signals are very clear.	❍	❍	❍	❍	❍	❍

Q6.2 Any comments? (optional)

____

Q7.1 So that we can understand your answers better, we have just a few questions about YOUR musical preferences and experience.
How much do you like music?
- ❍ Really hate
- ❍ Dislike
- ❍ Neutral
- ❍ Like
- ❍ Really love

Q7.2 How much do you like jazz?
- ❍ Really hate
- ❍ Dislike
- ❍ Neutral
- ❍ Like
- ❍ Really love

Q7.3 How much do you know about music?
- ❍ Nothing at all
- ❍ A little
- ❍ Moderate knowledge
- ❍ A lot
- ❍ Expert

Q7.4 How much do you know about jazz?
- ❍ Nothing at all
- ❍ A little
- ❍ Moderate knowledge
- ❍ A lot
- ❍ Expert

Q7.5 For how many years did you engage in regular, daily practice/playing of a musical instrument (including voice)?
- ❍ 0 years
- ❍ up to 2 years
- ❍ 2 - 5 years
- ❍ 5 - 10 years
- ❍ 10 or more years

Q7.6 At the peak of your interest, for how many hours per day did you practice/play on your primary instrument?
- 0 hours
- up to 1 hour
- 1 - 2 hours
- 2 - 4 hours
- 4 or more hours

Q7.7 How much experience do you have in improvising musically?
- None
- A little
- Some
- A lot
- A great deal

Q7.8 How much experience do you have playing in music duos (two performers)? (this can include voice)
- None
- A little
- Some
- A lot
- A great deal

Q7.9 How much experience do you have playing in larger ensembles (more than two performers)? (this can include voice)
- None
- A little
- Some
- A lot
- A great deal

Q7.10 How much experience do you have playing in jam sessions (including voice)?
- None
- A little
- Some
- A lot
- A great deal

Q7.11 In the past twelve months, how many live music events have you attended as an audience member?
- ❍ 0 events
- ❍ 1 - 2 events
- ❍ 3 - 6 events
- ❍ 7 - 10 events
- ❍ 11 or more events

Q7.12 In the past twelve months, how many live jazz events have you attended as an audience member?
- ❍ 0 events
- ❍ 1 - 2 events
- ❍ 3 - 6 events
- ❍ 7 - 10 events
- ❍ 11 or more events

Q7.13 How many years of formal training in music theory have you had?
- ❍ 0 years
- ❍ up to 2 years
- ❍ 2 - 5 years
- ❍ 5 - 10 years
- ❍ 10 or more years

Q7.14 How many years of formal training on a musical instrument (including voice) have you had?
- ❍ 0 years
- ❍ up to 2 years
- ❍ 2 - 5 years
- ❍ 5 - 10 years
- ❍ 10 or more years

Q7.15 How long do you listen attentively to music per day?
- ❍ I never listen
- ❍ 30 mins or less
- ❍ 30 - 60 mins
- ❍ 60 - 90 mins
- ❍ 90 mins or more

Q7.16 How long do you listen attentively to jazz per day?
❍ I never listen
❍ 30 mins or less
❍ 30 - 60 mins
❍ 60 - 90 mins
❍ 90 mins or more

Q7.17 How often do you talk or write about music?
❍ Never
❍ Rarely
❍ Sometimes
❍ Regularly
❍ Frequently

Q7.18 How often do you talk or write about jazz?
❍ Never
❍ Rarely
❍ Sometimes
❍ Regularly
❍ Frequently

Q7.19 Which of these instruments do you now play on a regular basis? (select all that apply)

- ❑ Saxophone
- ❑ Clarinet
- ❑ Oboe
- ❑ Bassoon
- ❑ Flute
- ❑ Piccolo
- ❑ Shakuhachi
- ❑ Harmonica
- ❑ Didgeridoo
- ❑ Trumpet
- ❑ French Horn
- ❑ Tuba
- ❑ Trombone
- ❑ Guitar (acoustic or electric)
- ❑ Bass guitar
- ❑ Violin
- ❑ Viola
- ❑ Cello
- ❑ Double Bass
- ❑ Banjo
- ❑ Ukelele
- ❑ Sitar
- ❑ Harp
- ❑ Kora
- ❑ Sarod
- ❑ Erhu
- ❑ Koto
- ❑ Oud
- ❑ Bazouki
- ❑ Uhadi
- ❑ Santar
- ❑ Drums
- ❑ Orchestral percussion instruments
- ❑ Xylophone
- ❑ Marimba
- ❑ Tabla
- ❑ Steel pan
- ❑ I play the following instrument(s) ____________________
- ❑ I do not play any musical instruments
- ❑ Piano
- ❑ Organ
- ❑ Harpsichord
- ❑ Electronic Keyboard
- ❑ Harmonium
- ❑ Accordion
- ❑ Voice

Q7.20 Which of these instruments have you ever played on a regular basis? (select all that apply)

- ❑ Saxophone
- ❑ Clarinet
- ❑ Oboe
- ❑ Bassoon
- ❑ Flute
- ❑ Piccolo
- ❑ Shakuhachi
- ❑ Harmonica
- ❑ Didgeridoo
- ❑ Trumpet
- ❑ French Horn
- ❑ Tuba
- ❑ Trombone
- ❑ Guitar (acoustic or electric)
- ❑ Bass guitar
- ❑ Violin
- ❑ Viola
- ❑ Cello
- ❑ Double Bass
- ❑ Banjo
- ❑ Ukelele
- ❑ Sitar
- ❑ Harp
- ❑ Kora
- ❑ Sarod
- ❑ Erhu
- ❑ Koto
- ❑ Oud
- ❑ Bazouki
- ❑ Uhadi
- ❑ Santar
- ❑ Drums
- ❑ Orchestral percussion instruments
- ❑ Xylophone
- ❑ Marimba
- ❑ Tabla
- ❑ Steel pan
- ❑ I play the following instrument(s) ____________________
- ❑ I do not play any musical instruments
- ❑ Piano
- ❑ Organ
- ❑ Harpsichord
- ❑ Electronic Keyboard
- ❑ Harmonium
- ❑ Accordion
- ❑ Voice

Q7.21 Which genre(s) of music do you play? (select all that apply)
- ❑ Rock/pop
- ❑ Jazz
- ❑ Classical
- ❑ Folk
- ❑ Other ____________________
- ❑ None

Q7.22 Which genre(s) of music do you choose to listen to regularly? (select all that apply)
- ❑ Rock/pop
- ❑ Jazz
- ❑ Classical
- ❑ Folk
- ❑ Other ____________________
- ❑ None

Q8.1 Great. We're almost done. We have a very few questions about your experience of this survey.

Q8.2 How did you hear about this study?
- ❍ The researchers running this study sent me the link
- ❍ Someone else I know sent me the link
- ❍ Someone else I don’t know sent me the link
- ❍ I found the link on a social networking site (e.g., Facebook)
- ❍ I found the link on a fan forum
- ❍ Some other way ____________________

Q8.3 How long did it take to answer all the questions?
- ❍ less than 1 hour
- ❍ 1 - 2 hours
- ❍ 2 hours or more

Q8.4 How easy was it to answer the questions?
- ❍ Very easy
- ❍ Somewhat easy
- ❍ Neither easy nor hard
- ❍ Somewhat hard
- ❍ Very hard

Q8.5 How much did you enjoy the experience?
- ❍ Not at all
- ❍ A little
- ❍ Somewhat
- ❍ A lot
- ❍ A great deal

Q8.6 Where did you fill in the questionnaire?
- ❍ At home
- ❍ At work
- ❍ Somewhere else ____________________

Q8.7 Were you alone the whole time?
- ❍ Yes
- ❍ No

Q8.8 Did you take breaks while answering the questions?
- ❍ Yes
- ❍ No

Q8.9 Do you have any guesses or thoughts about who was playing?
- ❍ No
- ❍ Yes ____________________

Q8.10 Is there anything else you would like us to know about your experience answering the questions?

Q9.1 Finally, to finish up we have a few questions about how you identify yourself, just for statistical purposes.I identify myself as
- ❍ Male
- ❍ Female

Q9.2 I classify myself as (select all that apply): (different countries and groups use different labels, so please add your own if needed)
- ❑ Arab
- ❑ Asian/Pacific Islander
- ❑ Black
- ❑ Caucasian/White
- ❑ Hispanic
- ❑ Indigenous or Aboriginal
- ❑ Latino
- ❑ Multiracial
- ❑ Other ____________________
- ❑ Would rather not say

Q9.3 The country I reside in is

❍ Please select below...

UNITED STATES	Central African Republic	Grenada	Macedonia	Poland	Togo
UNITED KINGDOM	Chad	Guatemala	Madagascar	Portugal	Tonga
Afghanistan	Chile	Guinea	Malawi	Qatar	Trinidad and Tobago
Albania	China	Guinea-Bissau (Bissau) (AF)	Malaysia	Romania	Tunisia
Algeria	Colombia	Guyana	Maldives	Russian Federation	Turkey
Andorra	Comoros	Haiti	Mali	Rwanda	Turkmenistan
Angola	Congo	Honduras	Malta	Saint Kitts and Nevis	Tuvalu
Antigua and Barbuda	Congo, Democratic Republic of	Hungary	Marshall Islands	Saint Lucia	Uganda
Argentina	Costa Rica	Iceland (79)	Mauritania	Saint Vincent and the Grenadines	Ukraine
Armenia	Cote d'Ivoire/Ivory Coast	India (80)	Mauritius	Samoa	United Arab Emirates
Australia	Croatia	Indonesia	Mexico	San Marino	United Kingdom
Austria	Cuba	Iran	Micronesia	Sao Tome and Principe	United States
Azerbaijan	Cyprus	Iraq	Moldova	Saudi Arabia	Uruguay
Bahamas	Czech Republic	Ireland	Monaco	Senegal	Uzbekistan
Bahrain	Denmark	Israel	Mongolia	Serbia	Vanuatu
Bangladesh	Djibouti	Italy	Montenegro	Seychelles	Vatican City
Barbados	Dominica	Jamaica	Morocco	Sierra Leone	Venezuela
Belarus	Dominican Republic	Japan	Mozambique	Singapore	Vietna
Belgiu	East Timor	Jordan	Namibia	Slovakia	Yemen
Belize	Ecuador	Kazakstan	Nauru	Slovenia	Zambia
Benin	Egypt	Kenya	Nepal	Solomon Islands	Zimbabwe
Bhutan	El Salvador	Kiribati	Netherlands	Somalia	Other
Bolivia	Equatorial Guinea	Korea, North	New Zealand	South Africa	
Bosnia and Herzegovina	Eritrea	Korea, South	Nicaragua	Spain	
Botswana	Estonia	Kuwait	Niger	Sri Lanka	
Brazil	Ethiopia	Kyrgyzstan	Nigeria	Sudan	
Brunei	Fiji	Laos	Norway	Suriname	
Bulgaria	Finland	Latvia	Oman	Swaziland	
Burkina Faso	France	Lebanon	Pakistan	Sweden	
Burma/Myanmar	Gabon	Lesotho	Palau	Switzerland	
Burundi	Gambia	Liberia	Panama	Syria	
Cambodia	Georgia	Libya	Papua New Guinea	Taiwan	
Cameroon	Germany	Liechtenstein	Paraguay	Tajikistan	
Canada	Ghana	Lithuania	Peru	Tanzania	
Cape Verde	Greece	Luxembourg	Philippines	Thailand	

Q9.4 The highest level of education that I have completed is
- ❍ Less than High School
- ❍ High School / GED
- ❍ Undergraduate / Bachelors Degree (including performance)
- ❍ Masters Degree (including performance)
- ❍ Doctoral Degree (including performance)
- ❍ Other Professional Degree (e.g., JD, MD)
- ❍ Other ____________________
- ❍ Would rather not say

Q9.5 On a scale of 1 to 5, my comprehension of the English language is
- ❍ 1 Poor
- ❍ 2
- ❍ 3
- ❍ 4
- ❍ 5 Excellent

Q9.6 My annual household salary (including bonuses and commissions) in U.S. dollars is
- ❍ $0 - $50,000
- ❍ $50,001 - $100,000
- ❍ $100,001 - $150,000
- ❍ $150,001+
- ❍ Would rather not say

Q10.1 That was the last question. Thank you very much for participating in our study! Click NEXT to proceed to a different website for entering your email address; this will allow you to be sent your Amazon.com gift code. There you can also leave any comments and let us know if you're interested in participating in any future studies. Thanks again!
